# Interleukin-13 receptor alpha 2 cooperates with EGFRvIII signaling to promote glioblastoma multiforme

**DOI:** 10.1038/s41467-017-01392-9

**Published:** 2017-12-04

**Authors:** Jennifer P. Newman, Grace Y. Wang, Kazuhiko Arima, Shou P. Guan, Michael R. Waters, Webster K. Cavenee, Edward Pan, Edita Aliwarga, Siao T. Chong, Catherine Y. L. Kok, Berwini B. Endaya, Amyn A. Habib, Tomohisa Horibe, Wai H. Ng, Ivy A. W. Ho, Kam M. Hui, Tomasz Kordula, Paula Y. P. Lam

**Affiliations:** 1 Laboratory of Cancer Gene Therapy, Cellular and Molecular Research Division, Humphrey Oei Institute of Cancer Research, National Cancer Centre, 11, Hospital Drive, Singapore, 169610 Singapore; 2 Department of Biomolecular Sciences, Saga Medical School, Saga, 840-8502 Japan; 3 School of Medicine, Virginia Commonwealth University, Richmond, VA 23298 USA; 4 Ludwig Institute for Cancer Research, San Diego, CA 92093 USA; 5 Department of Neurology and Neurotherapeutics, University of Texas Southwestern Medical Center, Dallas, TX 75390 USA; 6 School of Medical Science, Griffith Health Institute, Griffith University, Southport, 4222 Queensland Australia; 7 Department of Neurology and Neurotherapeutics, University of Texas Southwestern Medical Center and the North Texas VA Medical Center, Dallas, 75390 USA; 8 Department of Pharmacoepidemiology, Kyoto University School of Public Health, Kyoto, 606-8501 Japan; 9 National Neuroscience Institute, 11 Jalan Tan Tock Seng, Singapore, 308433 Singapore; 10 Bek Chai Heah Laboratory of Cancer Genomics, Cellular and Molecular Research Division, Humphrey Oei Institute of Cancer Research, National Cancer Centre, Singapore, 169610 Singapore; 11 Cancer and Stem Cells Biology Program, Duke-NUS Graduate Medical School, 8 College Road, Singapore, 169857 Singapore; 12 Department of Biochemistry, Yong Loo Lin School of Medicine, National University of Singapore, 8 Medical Dr, Singapore, 117596 Singapore; 13 Institute of Molecular and Cell Biology, A*STAR, Proteos, 61 Biopolis Dr, Singapore, 138673 Singapore; 14 Department of Physiology, Yong Loo Lin School of Medicine, National University of Singapore, 2 Medical Drive, MD9, Singapore, 117593 Singapore; 15 Children’s Hospital Oakland Research Institute, 5700 Martin Luther King Jr. Way, Oakland, CA 94609 USA; 16 National Neuroscience Institute, 11 Jalan Tan Tock Seng, Singapore, 308433 Singapore

## Abstract

The interleukin-13 receptor alpha2 (IL-13Rα2) is a cancer-associated receptor overexpressed in human glioblastoma multiforme (GBM). This receptor is undetectable in normal brain which makes it a highly suitable target for diagnostic and therapeutic purposes. However, the pathological role of this receptor in GBM remains to be established. Here we report that IL-13Rα2 alone induces invasiveness of human GBM cells without affecting their proliferation. In contrast, in the presence of the mutant EGFR (EGFRvIII), IL-13Rα2 promotes GBM cell proliferation in vitro and in vivo. Mechanistically, the cytoplasmic domain of IL-13Rα2 specifically binds to EGFRvIII, and this binding upregulates the tyrosine kinase activity of EGFRvIII and activates the RAS/RAF/MEK/ERK and STAT3 pathways. Our findings support the “To Go or To Grow” hypothesis whereby IL-13Rα2 serves as a molecular switch from invasion to proliferation, and suggest that targeting both receptors with STAT3 signaling inhibitor might be a therapeutic approach for the treatment of GBM.

## Abstract (web summary)

Interleukin-13 receptor alpha 2 is highly expressed in glioblastoma multiforme but its role in this malignancy is unclear. Here the authors show that this receptor interacts with mutant EGFR, stimulating its kinase activity, thus inducing proliferation.

## Introduction

Glioblastomas (GBM) are primary brain tumors that are among the most lethal of all cancers. The prognosis for patients diagnosed with these tumors remains dismal, with a median survival rate of less than 15 months, and the 5-year median survival rate of <3%^1^. The extent of crosstalk between key signaling pathways, in the context of GBM, is still poorly understood.

Two commonly expressed tumor antigens in GBM include the epidermal growth factor receptor mutant (EGFRvIII) and the interleukin-13 receptor alpha 2 (IL-13Rα2), which are potential targets for the treatment of GBM^2,3^. Overexpression of EGFRvIII in human GBM typically ranges from 25 to 81%^4,5^. Interestingly, EGFRvIII is rare in low-grade glioma. Thus, its high occurrence in high-grade glioma supports its essential role in the progression of GBM^6^. The EGFRvIII mutant is generated from an in-frame deletion of 267 amino acids from the extracellular domain of the wild-type (wt) EGFR^7^. As a consequence, its tyrosine kinase is constitutively activated, which accounts for its oncogenic potential.

The IL-13Rα2 receptor is a 42-kDa monomeric receptor with high binding affinity to IL-13, but not IL-4^8^. IL-13 plays an important role in epithelial tissue repair and this effect is mediated through the autocrine release of EGF and the subsequent activation of EGFR^9^. Further, inhibition of the EGFR tyrosine kinase activity by tyrphostin AG1478 increases IL-13 release after an injury, suggesting a negative feedback between EGFR and IL-13. Apart from IL-13, another ligand of IL-13Rα2 is the chitinase 3-like 1 (CHI3L1, also known as YKL-40/BRP-39)^10^. This is a secreted glycoprotein of ~40 kDa in size, which has been implicated in inflammatory diseases, tissue remodeling, and cancer progression^11^. IL-13/IL-13Rα2 interaction does not lead to activation of the JAK/STAT6 pathway; thus, it has been regarded as a decoy receptor^12^. Interestingly, IL-13 was shown to signal through the IL-13Rα2 receptor in an AP-1-dependent manner to transactivate the transforming growth factor beta-1 (TGFβ-1) promoter in macrophages and monocytes^13^. This increase in TGFβ-1 levels contributes to lung fibrosis. The cytoplasmic tail of IL-13Rα2 has been shown to inhibit IL-4-mediated signaling^14^. The expression of IL-13Rα2 is restricted to the testis and is completely absent or low in other normal somatic tissues^15,16^. In contrast, elevated expression of IL-13Rα2 has been found in ~75% of GBM patients^16–20^. The levels of IL-13Rα2 expression correlates with tumor grades of astrocytomas, and is a prognostic indicator of poor patient survival^3,21^. Elevated expression of IL-13Rα2 was also detected in primary tumor samples from 61% of brainstem glioma^18^ and 83% of pediatric brain tumors, mainly the high-grade astrocytomas^19^. Apart from high-grade gliomas, the receptor has been reported to be overexpressed in several types of human tumors, including head and neck cancer^22^, kidney cancer^23^, prostate cancer^24^, ovarian cancer^25^, adrenocortical carcinoma^26^, clear cell renal cell carcinoma^27^, and Kaposi’s sarcoma^28^. The sharp contrast in expression levels has made it an excellent cancer-specific antigen to develop various targeted therapeutic strategies, including IL-13 conjugated bacterial toxins and modifying oncolytic virus and cytolytic T-cells, such that they express the IL-13 moiety^29^. Recently, a patient suffering from multifocal glioblastoma has also shown regression of all intracranial and spinal tumors after treatment with IL-13Rα2 specific CAR T cells^30^. Despite the various IL-13Rα2 targeted approaches and promising results obtained from clinical trials, little is known about the role of IL-13Rα2 in GBM development and progression.

IL-13Rα2 was identified as one of the downstream targets of EGFRvIII^31^. Interestingly, expression of EGFRvIII and IL-13Rα2 has been reported in 84% and 79% of primary GBM patient tumors, respectively^32^. Therefore, we hypothesized that IL-13Rα2 might cooperate with EGFRvIII and contribute to GBM progression. Our data demonstrate that in the absence of EGFRvIII, the overexpression of IL-13Rα2 promotes GBM invasion but not proliferation. In contrast, in the presence of the mutant EGFR (EGFRvIII), IL-13Rα2 interacts with EGFRvIII to promotes GBM growth through upregulation of EGFRvIII tyrosine kinase activities and subsequently the RAS/RAF/MEK/ERK and STAT3 pathways.

## Results

### IL-13Rα2 promotes invasion but not cell proliferation

We analyzed *IL-13Rα2* mRNA expression from primary tumor patient samples obtained from the Repository for the Molecular Brain Neoplasia Data (REMBRANDT) of the National Cancer Institute. The patient survival outcome correlated significantly with the levels of IL-13Rα2 mRNA expression. There were 121 tumors (red) with increased levels, 107 tumors (yellow) with intermediate levels and 115 tumors (green) without detectable levels (Supplementary Fig. 1a). Enhanced IL-13Rα2 levels were confirmed in established GBM cell lines when compared to primary human astrocytes (Supplementary Fig. 1b).

**Fig. 1:**
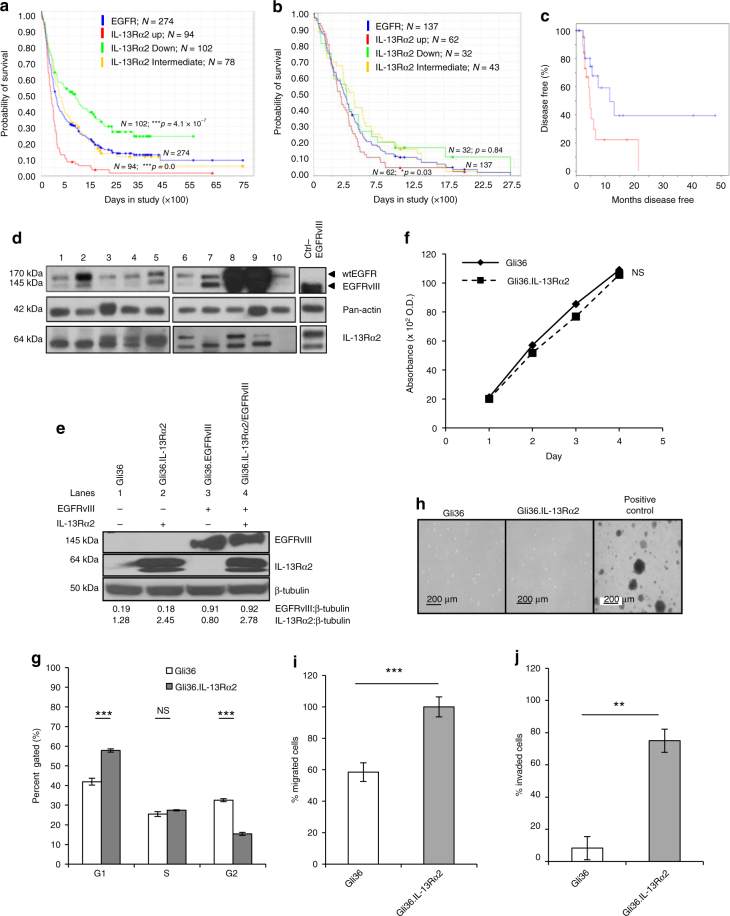
GBM patients co-expressing EGFR and IL-13Rα2 correlate to poor survival where the overexpression of IL-13Rα2 alone leads to enhance cell migration but not proliferation. Kaplan−Meier survival analysis of **a** all gliomas patients; **b** GBM patients from REMBRANDT database from National Cancer Institute (USA). Patients overexpressing EGFR mRNA by 2-fold (blue) with high (red), intermediate (yellow) and low (green) levels of IL-13Rα2 expression were shown. The log-rank *p*-values were indicated. **c** Kaplan−Meier survival plots for patients expressing high YKL-40 mRNA levels TCGA. High IL-13Rα2 expression group (red) and low IL-13Rα2 expression group (blue) were determined by aggregating all patients whose *z*-score normalized expression was above or below 0, respectively (Log-rank test *p*-value = 0.0374). Immunoblotting analysis showed the expression of EGFR and IL-13Rα2 protein levels were determined from **d** a panel of 10 patient-derived GBM **e** and the isogenic cell lines generated from Gli36 glioma cells. Pan-actin or β tubulin served as internal loading controls. **f** Cell proliferation and **g** Cell cycle analysis were performed with Gli36 and Gli36.IL-13Rα2 cells **h** Soft agar colony formation assay was performed, Gli36.EGFRvIII was used as a positive control. **i** In vitro migration and **j** invasion assays were determined in Gli36 and Gli36.IL-13Rα2 cells. All data are represented as mean ± SEM, unpaired *t*-test **p < 0.01; ***p < 0.001; NS not significant

Next, we analyzed all gliomas patients in which EGFR mRNA was overexpressed by twofold (Fig. 1a; blue). Of the 274 patients evaluated, 94 patients expressing high levels of IL-13Rα2 (red) showed statistically significant decreased survival rate compared to 102 patients with low levels of IL-13Rα2 (green). Similar findings were observed in GBM patients, albeit the total number of patients was smaller. Of the 137 GBM patients with twofold overexpression of EGFR mRNA, 62 patients with high levels of IL-13Rα2 (red) had a poor survival outcome when compared to 32 patients with low levels of IL-13Rα2 (green) (Fig. 1b; log-rank *p*-value = 0.03). The association between EGFR and IL-13Rα2 was calculated by chi-square analysis (Supplementary Fig. 1c; *p*-value 0.0399; Fisher’s exact test = 0.0328). Since both IL-13 and CHI3L1 signal via IL-13Rα2, we subsequently analyzed IL-13Rα2-dependent survival of patients expressing either high levels of IL-13 or CHI3L1 (The Cancer Genome Atlas, TCGA datasets). Kaplan−Meier survival analysis identified that high level of IL-13Rα2 with high levels of CHI3L1 (Fig. 1c) or IL-13 (Supplementary Fig. 1d) had a significant negative impact on patient survival. The expression of IL-13Rα2 and EGFR proteins was confirmed in primary tumor samples from Singaporean patients with GBM. With the exception of one sample, all GBM samples expressed IL-13Rα2 to varying degree (~64-kDa and detectable levels of both wtEGFR (~170kDa) and mutant EGFRvIII (~145kDa) proteins (Fig. 1d), suggesting that these receptors may play a role in gliomagenesis.

In an attempt to delineate the functional relationship between IL-13Rα2 and EGFRvIII in human gliomas, isogenic human glioma cell lines that expressed either IL-13Rα2, EGFRvIII, both or null were generated to recapitulate the heterogeneous nature of the human GBM tumors. The expression of IL-13Rα2 and EGFR in glioma cell lines used in our studies was first determined (Supplementary Fig. 2a, b, respectively). Human glioma Gli36 cells were chosen to constitutively express IL-13Rα2 alone, EGFRvIII or both receptors as confirmed by immunoblotting analysis and denoted as Gli36.IL-13Rα2, Gli36.EGFRvIII and Gli36.IL-13Rα2/EGFRvIII, respectively (Fig. 1e). Overexpression of IL-13Rα2 did not result in detectable difference in the proliferation kinetics (Fig. 1f), S-phase of cell cycle profile (Fig. 1g) nor in the number of soft agar colonies formed (Fig. 1h), thus, confirming that overexpression of IL-13Rα2 alone could not confer growth advantage. However, the expression of IL-13Rα2 could induce an increase in cell migration (Fig. 1i) and cell invasion (Fig. 1j). Taken together, these findings demonstrated a possible role of IL-13Rα2 in the migration and invasion of GBM cells, in vitro.

### IL-13Rα2 induces EMT-like changes favoring GBM invasion

**Fig. 2:**
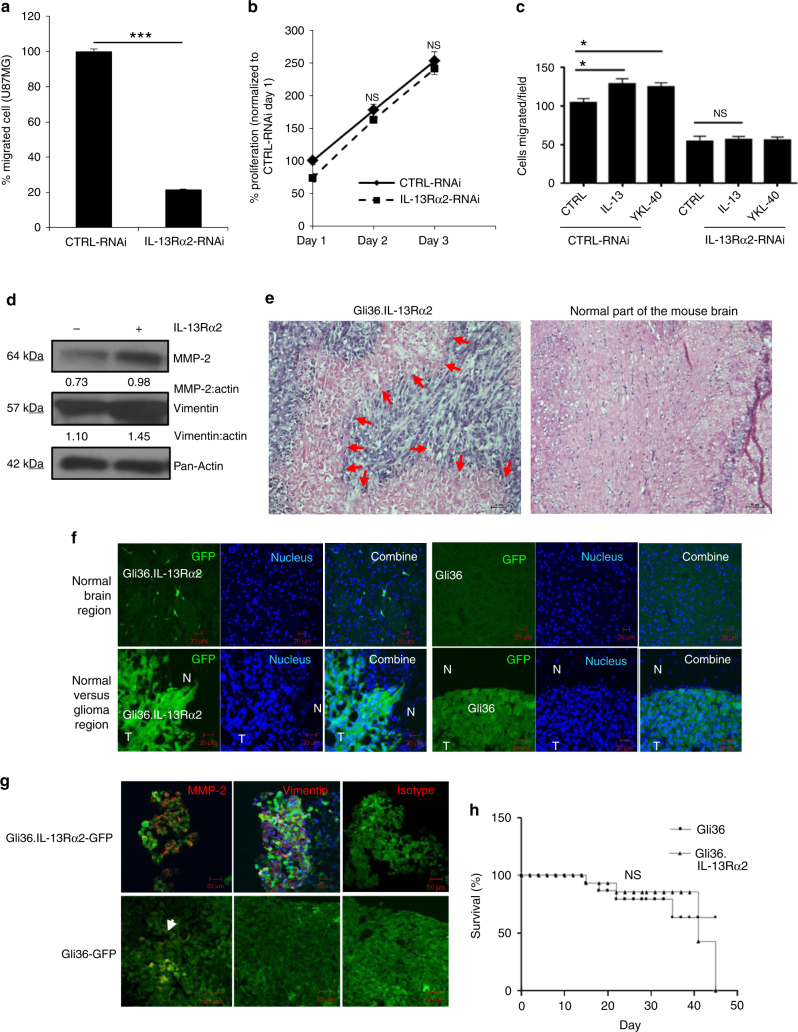
Ectopic expression of IL-13Rα2 promotes glioma invasion. **a** In vitro migratory capacity of control and IL-13Rα2-RNAi treated U87MG cells was determined. Percent of migrated cells was normalized to CTRL-RNAi. **b** U87MG cells were transfected with non-specific siRNA (CTRL-RNAi) or IL-13Rα2 specific siRNA (IL-13Rα2-RNAi). Cell proliferation was subsequently determined, and the percent of proliferation was normalized to CTRL-RNAi day 1. **c** In vitro migratory capacity of control and IL-13Rα2-RNAi treated U87MG cells, stimulated with 1 µg ml^−1^ YKL40 or 20 ng ml^−1^ IL-13 for 18 h, was determined using wound-healing migration assay. All data are represented as mean ± SEM. Unpaired *t*-test *p < 0.05; ***p < 0.001; NS not significant. **d** Immunoblotting experiment showing upregulation of MMP-2, and vimentin in Gli36.IL-13Rα2 cells. Densitometry quantification was done for the indicated proteins by normalizing to pan-actin as the internal loading control. Ratios were indicated below each blot. **e** Mice were implanted with either Gli36-GFP or Gli36.IL-13Rα2-GFP cells intracranially. Tumors were collected from representative mice implanted with Gli36.IL-13Rα2-GFP showed the invasive phenotype (left panel) when compared to the contralateral normal brain parenchyma (right panel) by haematoxylin and eosin (H&E) staining. Red arrows indicated glioma tumor at the invasive front **f** Representative images of mouse brain transplanted with Gli36- GFP cells and Gli36-IL-13Rα2-GFP, counterstained with DAPI (blue). The top panel shows the contralateral hemisphere; bottom panel shows tumor-bearing hemisphere of the mouse brain (N, normal; T, tumor). Scale bar, 50 μm. **g** Immunofluorescence red staining of MMP-2, vimentin or isotypic control in mice bearing either Gli36.IL-13Rα2-GFP or Gli36-GFP. Scale bar, 50 μm. **h** Kaplan−Meier survival curves of mice bearing Gli36 and Gli36.IL-13Rα2 tumors. NS not significant

To further confirm the role of IL-13Rα2 in cell migration and invasion, targeted knockdown of IL-13Rα2 was performed in human glioma U87MG cells expressing endogenous IL-13Rα2, and the siRNA knockdown efficiency was confirmed by immunoblot analysis (Supplementary Fig. 2c). Knockdown of IL-13Rα2 expression significantly reduced glioma migration in U87MG (Fig. 2a), but did not affect cell proliferation (Fig. 2b). Similar findings were observed in another human glioma cell line U251MG, which has a high endogenous expression of IL-13Rα2 (Supplementary Fig. 2d). In these cells, targeted knockdown of IL-13Rα2 did not affect cell viability (Supplementary Fig. 2e), but reduced invasiveness (Supplementary Fig. 2f). In addition, knockdown of IL-13Rα2 inhibited basal, and IL-13- and CHI3L1/YKL40-induced migration of both U87MG (Fig. 2c) and U251MG cells (Supplementary Fig. 2g).

Matrix metalloproteases (MMPs)-2 and vimentin are reported to be associated with GBM invasion ^33,34^. Ectopic expression of IL-13Rα2 resulted in enhanced MMP-2 and vimentin expression when compared to parental Gli36 cells (Fig. 2d). Histological analysis of IL-13Rα2-positive glioma tumor brain sections showed infiltrating tumor cells invading into normal brain parenchymal (Fig. 2e; as indicated by the red arrow). Similar findings were observed using glioma cells transduced with pWPT-GFP lentiviral vectors expressing the green fluorescence proteins (GFP). Expression of IL-13Rα2 in Gli36.IL-13Rα2-GFP was not altered by the infection (Supplementary Fig. 2h). Intracranial transplantation of Gli36.IL-13Rα2-GFP cells showed the invasive property of the cells, which was absent in Gli36-GFP cells (Fig. 2f). Consistent with in vitro findings, MMP-2 and vimentin were also upregulated in IL-13Rα2-positive tumors, as compared to control (Fig. 2g). No significant difference in survival was observed in mice intracranially transplanted with Gli36 and Gli36.IL-13Rα2 cells (Fig. 2h). The corresponding histopathological hematoxylin and eosin staining were shown in Supplementary Fig. 3f. Taken together, these data demonstrate that overexpression of IL-13Rα2 alone could promote cell migration and invasion, but not proliferation.

### IL-13Rα2 induces proliferation in the presence of EGFRvIII

**Fig. 3:**
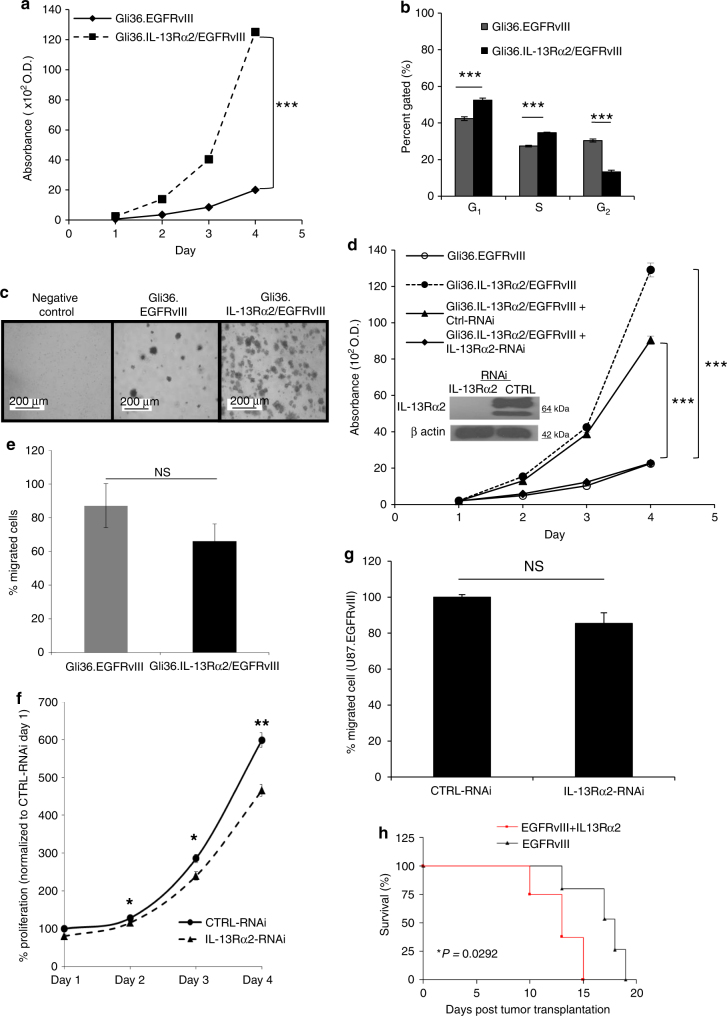
Co-expression of IL-13Rα2 and EGFRvIII enhances glioma transformation. **a** Cell proliferation of Gli36.EGFRvIII and Gli36.IL-13Rα2/EGFRvIII. **b** The cell cycle profile was compared between Gli36.EGFRvIII and Gli36.IL-13Rα2/EGFRvIII using flow cytometry analysis. **c** Soft agar colony formation assay of the indicated cells was performed, including the non-transforming Gli36 cells as a negative control. **d** IL-13Rα2 silencing (as validated by immunoblot presented as an insert) significantly reduced the proliferation of Gli36.IL-13Rα2/EGFRvIII cells to a level similar to the Gli36.EGFRvIII cells. **e** In vitro migratory capacity of Gli36.EGFRvIII and Gli36.IL-13Rα2/EGFRvIII cells. **f** U87MG.EGFRvIII cells were transfected with non-specific siRNA (CTRL-RNAi) or IL-13Rα2 specific siRNA (IL-13Rα2-RNAi). Cell proliferation was determined with CCK-8 assay in U87.EGFRvIII. The percent proliferation was normalized to CTRL-RNAi day 1. **g** In vitro migratory capacity of control and IL-13Rα2-RNAi treated U87MG.EGFRvIII was determined. Percent of migrated cells was normalized to CTRL-RNAi. All data are represented as mean ± SEM. ANOVA with Tukey's multiple comparison tests *p < 0.05; ***p < 0.001; NS not significant. **h** Kaplan−Meier survival curves of mice bearing Gli36.EGFRvIII and Gli36.IL-13Rα2/EGFRvIII tumors *p < 0.0292

Next, we ascertained the biological consequences of IL-13Rα2 expression in the presence of EGFRvIII. The results showed that human glioma cells co-expressing IL-13Rα2 and EGFRvIII exhibited a higher growth rate compared to EGFRvIII-positive cells (Fig. 3a). Cells expressing both receptors showed a marked reduction of G_2_/M cell population and a concomitant increase in G_0_/G_1_ and S populations compared to Gli36.EGFRvIII cells by FACS analysis (Fig. 3b). Cells co-expressing IL-13Rα2 and EGFRvIII exhibited increased anchorage-independence (Fig. 3c), which was approximately 3-fold higher than that exhibited by cells expressing EGFRvIII alone (Supplementary Fig. 3a). Targeted knockdown of IL-13Rα2 in Gli36.IL-13Rα2/EGFRvIII cells abolished the growth advantage (Fig. 3d), thus, indicating a specific gain-of-function associated with IL-13Rα2 in cell proliferation. However, no significant change was observed in the migratory potential of these two cell types (Fig. 3e).

Consistent with the earlier findings, targeted knockdown of endogenous IL-13Rα2 in U87MG.EGFRvIII cells as confirmed by immunoblot analysis (Supplementary Fig. 3b), resulted in significant reduced cell proliferation kinetics (Fig. 3f). There was a modest reduction in the migratory potential in IL-13Rα2 RNAi-treated cells when compared to the control cells, but the difference was statistically in significant (Fig. 3g). Similarly, targeted knockdown of IL-13Rα2, which was validated by immunoblot analysis (Supplementary Fig. 3c), in primary EGFRvIII-positive Mayo clinic derived GBM patient tumor, resulted in reduced cell proliferation (Supplementary Fig. 3d) and thus further confirmed the notion that IL-13Rα2 augmented cellular proliferation in the presence of EGFRvIII. However, there was no difference in cell migration (Supplementary Fig. 3e). More importantly, immunodeficient mice intracerebrally transplanted with Gli36.IL-13Rα2/EGFRvIII cells have significantly shorter lives compared to the Gli36.EGFRvIII group (*P = 0.029; Fig. 3h). Immunohistochemical staining of tissue from the two groups confirmed the expression of IL-13Rα2 and EGFR, respectively (Supplementary Fig. 3f). Thus, these data indicated that IL-13Rα2 cannot enhance proliferation alone, but instead promotes cellular proliferation in collaboration with the growth factor receptor EGFRvIII.

### IL-13Rα2 and EGFRvIII enhances activation of MAPK and STAT3

**Fig. 4:**
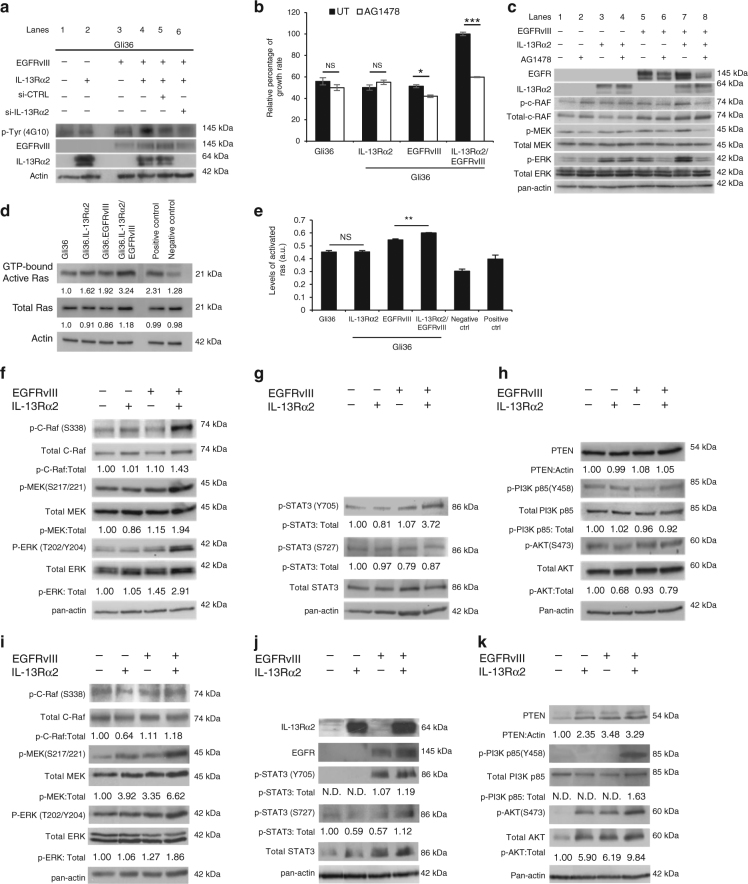
Oncogenic signaling of IL-13Rα2 increases tyrosine kinase activities and promote cell proliferation through activation of the RAS/RAF/MEK/ERK signaling cascade. **a** Cell lysates from Gli36, Gli36.IL-13Rα2, Gli36.EGFRvIII, and Gli36.EGFRvIII/IL-13Rα2 post-knockdown with scrambled or IL-13Rα2 siRNA were examined for the total levels of tyrosine phosphorylation using anti-phosphotyrosine antibodies. **b** CCK-8 proliferation assay was performed with and without 10 μM AG1478 treatments. All data are represented as mean ± SEM. Unpaired *t*-test ***p < 0.001 **c** Endogenous expression levels of the RAS/RAF/MEK/ERK signaling were examined in the indicated cells with and without AG1478 treatment. RAS activation was determined by either **d** Raf-1 RBD agarose beads pull-down assay or **e** ELISA assay. Endogenous protein expression levels of **f** Total and p-C-RAF, Total and p-MEK/p-ERK **g** Total and p-STAT3 **h** PTEN, total and p-PI3K p85α and total and p-AKT were examined in the indicated cells. Normal human astrocytes transfected with EGFRvIII, IL-13Rα2 or co-expressing both receptors were examined for endogenous expression levels of **i** Total and p-C-RAF, Total and p-MEK/p-ERK, **j** Total and p-STAT3, **k** PTEN, total and p-PI3K p85α and total and p-AKT. For all immunoblots, pan-actin served as internal loading controls, and band densitometry quantifications for the proteins were performed using ImageJ (NIH). The value derived from densitometry quantification is obtained from normalizing each of the signaling proteins against actin in a single experiment, and presented as a ratio of phosphorylate form over total protein. Each of these was performed at least two independent times

In an attempt to examine the mechanism of enhanced cellular proliferation induced by co-expression of IL-13Rα2 and EGFRvIII, the total tyrosine kinase activities of Gli36.IL-13Rα2/EGFRvIII and Gli36.EGFRvIII cells were examined by immunoblotting with anti-phosphotyrosine antibodies under serum starved conditions. The results showed that elevated tyrosine kinase activities was observed in cells co-expressing both receptors (Fig. 4a, lane 4) as compared to parental Gli36 cells (Fig. 4a, lane 1), and cells expressing either IL-13Rα2 or EGFRvIII (Fig. 4a lanes 2 or 3, respectively). Tyrosine kinase activities were significantly reduced in the presence of si-IL-13Rα2 (Fig. 4a, lane 6), when compared to si-CTRL (Fig. 4a, lane 5) or the untreated (Fig. 4a, lane 4). To further confirm that IL-13Rα2 could induce tyrosine kinase activities in EGFRvIII-positive cells, targeted silencing of endogenous IL-13Rα2 was performed in EGFRvIII-positive U251-E18 and primary GBM culture derived from patients. The results consistently showed that targeted silencing of IL-13Rα2 resulted in a significant reduction in tyrosine kinase activities when compared to naive or siCTRL controls (Supplementary Fig. 4a).

Next, we investigated the effect of enhanced tyrosine kinase activities on cell proliferation using a tyrosine kinase inhibitor, AG1478. The results demonstrated that AG1478 could significantly inhibit cell proliferation only in EGFRvIII-positive cells or in cells co-expressing EGFRvIII and IL-13Rα2. The relative growth rate of AG1478-treated Gli36.EGFRvIII cells, albeit reduced, was less dramatic compared to AG1478-treated Gli36.IL-13Rα2/EGFRvIII cells (Fig. 4b). No significant difference was observed in AG1478 pre-treated Gli36 or Gli36 overexpressing IL-13Rα2 (Fig. 4b). Similar findings were also observed in tetracyclin-induced EGFRvIII-positive U251-E18 (Supplementary Fig. 4b) and EGFRvIII-positive GBM patient-derived primary cells (Supplementary Fig. 4c).

Activation of ERK1/2 via EGFRvIII-MAPK is an essential pathway for enhanced proliferation of U87MG.EGFRvIII^35^. Therefore, we examined the effect of AG1478 inhibition on ERK1/2 activation in glioma cells co-expressing IL-13Rα2 and EGFRvIII. The results showed that activated MEK/ERK proteins were reduced to a greater extent in Gli36.IL-13Rα2/EGFRvIII, when compared to Gli36.EGFRvIII alone (Fig. 4c, compared lanes 8 vs. 6), as confirmed by semi-quantification of the corresponding protein levels (Supplementary Fig. 4d). Further, there was no significant difference in the STAT3 and PTEN pathways (Supplementary Fig. 4e, f, respectively), although a slight decrease in activated Akt was observed. Similar reduction in activated MEK/ERK proteins were also observed in tetracyclin-induced EGFRvIII-positive U251-E18 (Supplementary Fig. 4g) and EGFRvIII-positive GBM patient-derived primary cells (Supplementary Fig. 4h). Taken together, these data clearly demonstrated that cells co-expressing IL-13Rα2 and EGFRvIII have enhanced activated MEK/ERK activities, which is correlated to cell growth. These cells were also more sensitive to AG1478 treatment compared to EGFRvIII alone.

To further confirm that IL-13Rα2 could activate ERK1/2 via EGFRvIII-MAPK, we investigated whether MAPK activation was induced by Ras activation. Affinity pull-down assay showed that the level of GTP-bound Ras proteins was marked enhanced in glioma cells co-expressing IL-13Rα2 and EGFRvIII when compared to EGFRvIII alone or IL-13Rα2 alone (Fig. 4d). Quantitative Ras ELISA assay further confirmed that activated Ras activities was highest when both IL-13Rα2 and EGFRvIII were expressed (Fig. 4e). Affinity pull-down and ELISA assays performed in EGFRvIII-positive U251MG cells showed similar results (Supplementary Fig. 4i, j respectively). As a consequence of RAS activation, c-RAF (Ser338), activated MEK and ERK were significantly upregulated in serum starved, IL-13Rα2 and EGFRvIII co-expressed cells when compared to either EGFRvIII or IL-13Rα2 alone (Fig. 4f).

Immunoblotting analysis also showed that the levels of p-STAT3-Tyr705 were slightly enhanced in Gli36.EGFRvIII cells as compared to Gli36 or Gli36.IL-13Rα2 (Fig. 4g). The increase is markedly enhanced in Gli36.IL-13Rα2/EGFRvIII, while there was no notable difference in the levels of p-STAT3-S727 (Fig. 4g), and PTEN, the PI3K adaptor subunit p85α (Fig. 4h) among the various isogenic cells. Similar findings were observed in U251-E18 cells (Supplementary Fig. 4k−m). The finding that IL-13Rα2 could enhance MAPK and STAT3 signaling in the presence of EGFRvIII was further confirmed using primary GBM from Mayo clinic that expressed endogenous EGFRvIII and IL-13Rα2. Targeted knockdown of IL-13Rα2 in these cells resulted in significantly reduced levels of MAPK (Supplementary Fig. 4n) and STAT3 expressions (Supplementary Fig. 4o), while there was no significant change in the levels of PTEN/PI3K/AKT pathway (Supplementary Fig. 4p). In contrast, ectopic expression of IL-13Rα2 in the absence of EGFRvIII did not result in significant change in the levels of key proteins in MAPK, STAT3, and PI3K/AKT pathways (Fig. 4f−h, respectively). To assess the potential of IL-13Rα2/EGFRvIII on Raf-ERK1/2 signaling, we transiently transfected the normal human astrocytes cells with IL-13Rα2, EGFRvIII, or both receptors under the serum-free medium. The results showed that cells expressing both IL-13Rα2 and EGFRvIII exhibited a significant increase in the levels of c-RAF (Ser338), activated MEK and ERK (Fig. 4i), and STAT3 (Fig. 4j). There was no significant change in the levels of PTEN/PI3K pathway (Fig. 4k). Taken together, IL-13Rα2 could only promote cellular proliferation in the presence of EGFRvIII through the activation of the RAS/RAF/MEK/ERK and STAT3 signaling cascades.

### Cytoplasmic domains of IL-13Rα2 interacts with EGFRvIII

**Fig. 5:**
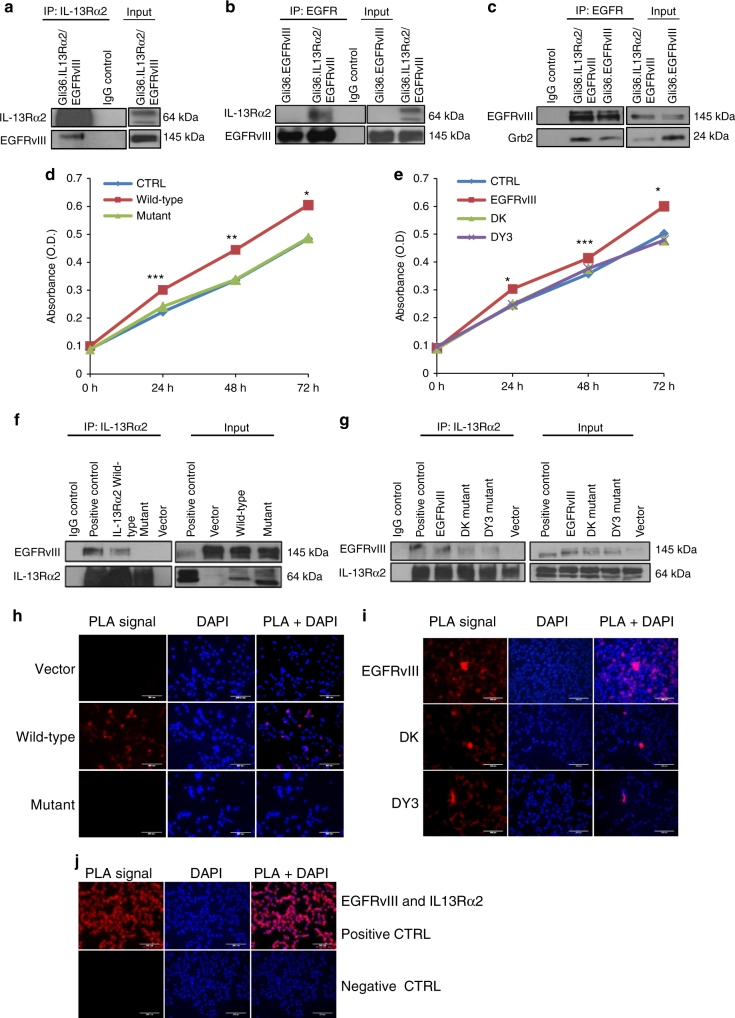
Deletion of the cytoplasmic domain of IL-13Rα2 resulted in a loss of physical interaction with EGFRvIII and enhanced proliferation is abolished. **a** Whole-cell lysates prepared from stable cell line Gli36.IL-13Rα2/EGFRvIII cells were used for immunoprecipitation with anti-IL-13Rα2 antibody, then immunoprobed with an anti-EGFR antibody. IgG served as control while unprecipitated extracts serve as input. **b** Similar cell lysates were reverse immunoprecipitated with anti-EGFR antibody, then immunoprobed with an anti-IL13Rα2antibody. Lysates from Gli36.EGFRvIII served as additional control **c** Gli36.IL-13Rα2/EGFRvIII cell lysates were immunoprecipitated with anti-EGFR antibody, then immunoprobed with anti-Grb antibody. To further examine the domains of interaction, IL-13Rα2 and EGFR mutants were used. Gli36.EGFRvIII cells were first transfected with pIRESneo2 (Vector), IL-13Rα2 full length (Wild-type) and IL-13Rα2 Cyt tail deleted constructs (Mutant) and then analyzed by **d** cell proliferation assay at the indicated time points, **f** co-immunoprecipitation, and **h** PLA assays. Findings were validated using Gli36.IL-13Rα2 cells transiently transfected with vector (CTRL), full length/wild-type EGFRvIII, DK, and DY3 mutants. **e** proliferation outputs, **g** co-immunoprecipitation, **i** and PLA assay were performed. **j** represent the corresponding positive and negative controls

Next, we examine whether IL-13Rα2 could directly associate with EGFRvIII. Co-immunoprecipitation using anti-IL-13Rα2 antibodies and reverse using EGFR antibodies in Gli36.IL-13Rα2/EGFRvIII cells confirmed the direction interaction of the two proteins (Fig. 5a, b, respectively). This is further supported by regions of overlap between IL-13Rα2 (red fluorescence) and EGFRvIII (green fluorescence) predominantly in the cytoplasm and some perinuclear regions of individual cells (Supplementary Fig. 5a). The interaction was confirmed in primary EGFRvIII-positive GBM patient tumors (Supplementary Fig. 5b) and U87MG.EGFRvIII expressing endogenous IL-13Rα2 (Supplementary Fig. 5c). Since IL-13Rα2 could promote oncogenesis via EGFRvIII signaling, therefore, we sought to determine whether the interaction of IL-13Rα2 with EGFRvIII led to enhanced association with adapter proteins. Co-immunoprecipitation results showed that the EGFRvIII association with adapter proteins, Grb2, was enhanced in the presence of IL-13Rα2 compared to without (Fig. 5c). This finding is consistent with the earlier observations where activated RAS was increased in glioma cells co-expressing IL-13Rα2 and EGFRvIII compared to EGFRvIII alone (Fig. 4d, e, Supplementary Fig. 4i, j).

To better understand how IL-13Rα2 interacts with EGFRvIII, we generated an IL-13Rα2 mutant deleted in the cytoplasmic tail (Supplementary Fig. 5d). Plasmid encoding wild-type or cytoplasmic tail mutant IL-13Rα2 was transfected into cells expressing Gli36.EGFRvIII and examined by cell proliferation assay and co-immunoprecipitation. Only wild-type IL-13Rα2 could promote cell proliferation (Fig. 5d) and interacted with EGFRvIII (Fig. 5f). In contrast, IL-13Rα2 mutant could not promote enhanced cell proliferation and failed to interact with EGFRvIII (Fig. 5d, f, respectively). Further, mutant IL-13Rα2 was incapable of further activating the MAPK, nor STAT and PI3K/AKT signaling cascades (Supplementary Fig. 6a−c, respectively).

To further confirm the interaction between IL-13Rα2 and EGFRvIII, similar experiments were performed in Gli36.IL-13Rα2 cells using a plasmid encoding the EGFRvIII cDNA and its mutant derivatives (Supplementary Fig. 5e). The kinase inactive EGFR mutant, DK mutant, does not possess any significant tyrosine phosphorylation ability. The DY3 mutant with point mutations at residues 1068, 1148, and 1173 has completely lost its ability to phosphorylate and confers a growth advantage in vivo^7^. Contrary to EGFRvIII, neither the DK nor DY3 mutants were capable of cooperating with IL-13Rα2 to enhance cell proliferation (Fig. 5e). Co-immunoprecipitation in Gli36.IL-13Rα2 cells transfected with the various EGFR constructs showed that the DK and DY3 mutant exhibited reduced binding ability when compared to EGFRvIII (Fig. 5g). Next, in situ proximity ligation assay (PLA) was employed to confirm protein interaction, which is visible as red fluorescence signals/PLA signals. Consistent with the co-immunoprecipitation results, PLA signals were detected in Gli36.EGFRvIII transfected with IL-13Rα2 but not the mutant or vector alone (Fig. 5h). Strong PLA signals were also detectable in Gli36.IL-13Rα2, transfected with EGFRvIII but these signals were significantly reduced when Gli36.IL-13Rα2 cells were transfected with the DK or DY3 mutants (Fig. 5i). Herein, stable cell line that constitutively expressed EGFRvIII and IL-13Rα2 served as positive control, while no detectable signal was observed with EGFR control (Fig. 5j), similar to additional negative controls such as IL-13Rα2 alone, rabbit PLA probe alone, and goat PLA probe alone. Taken together, these results demonstrated that enhanced cell proliferation was mediated through the association of the cytoplasmic tail of IL-13Rα2 with EGFRvIII.

### IL-13Rα2 promotes proliferation specifically with EGFRvIII

**Fig. 6:**
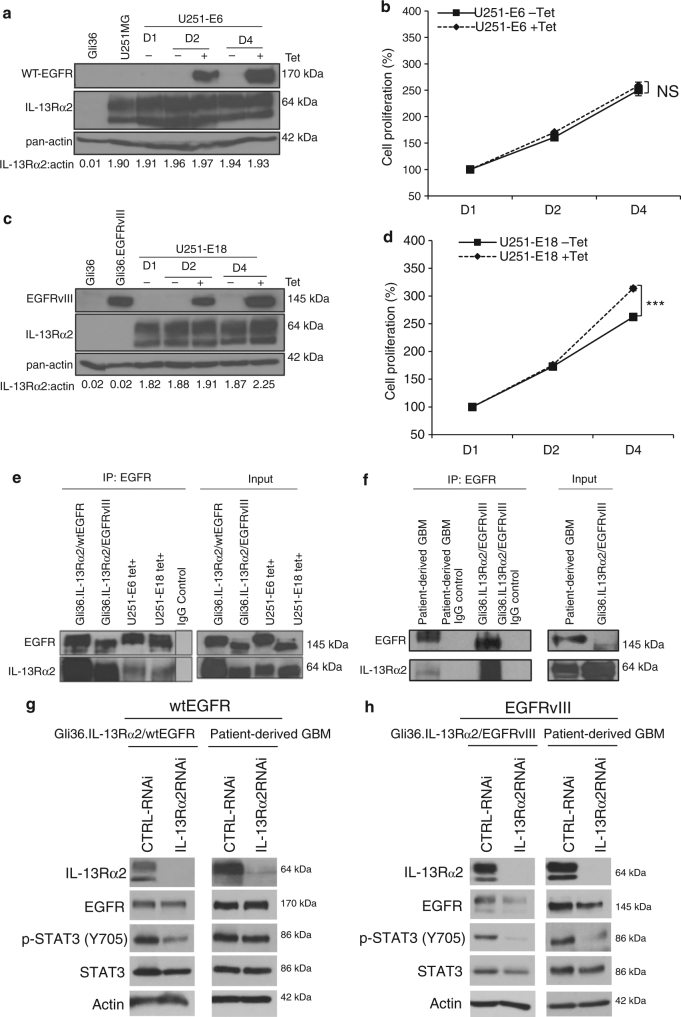
Enhanced cellular proliferation mediated by IL-13Rα2 is specific to EGFRvIII, and not WT EGFR. **a** U251-E6 or **c** U251-E18 cells were treated with or without tetracycline (Tet). At indicated time points, immunoblot analysis was carried out. Gli36, Gli36.EGFRvIII cell lysates were included as negative or positive controls for EGFRvIII, respectively. Growth kinetics of **b** U251-E6 and **d** U251-E18 was determined by CCK-8 assay. Percent cell viability was normalized to day 1 (without induction). All data are represented as mean ± SEM. Unpaired *t*-test ***p < 0.001, NS. not significant. **e** Co-immunoprecipitation was performed in stable cell lines Gli36.IL-13Rα2/wtEGFR as well as U251MG-E6 (i.e. wtEGFR) cells at 48 h post tetracycline induction with the indicated antibodies. Gli36.IL-13Rα2/EGFRvIII served as positive controls. **f** The interaction between endogenous wtEGFR and IL-13Rα2 was shown in primary wtEGFR-positive GBM patient tumor derived from Mayo clinic, and IgG served as positive and negative controls respectively. Knockdown of IL-13Rα2 in cell line or patient-derived GBM samples expressing **g** wtEGFR or **h** EGFRvIII

Next, we investigated whether IL-13Rα2 could provide a growth advantage in the presence of wtEGFR, using a tetracycline (Tet)-inducible wtEGFR (denoted as U251-E6) or mutant EGFRvIII (denoted as U251-E18) in the glioblastoma cell line U251MG. These cells expressed high endogenous levels of IL-13Rα2 at both the mRNA and protein levels^36^, which were also confirmed in Supplementary Fig. 2a, d, respectively. The induction of wtEGFR in U251-E6 cells did not appear to alter the levels of IL-13Rα2 protein (Fig. 6a) nor enhance proliferation (Fig. 6b). In contrast, the induction of EGFRvIII proteins in U251-E18 cells (Fig. 6c) significantly increased proliferation (Fig. 6d). Similarly, Gli36 cells co-expressing wtEGFR and IL-13Rα2 did not proliferate differently from Gli36.wtEGFR cells (Supplementary Fig. 7a). Cell cycle analysis revealed a corresponding accumulation of cells in the G_0_/G_1_ phase and the G_2_/M phase with no significant difference in proliferating the S-phase (Supplementary Fig. 7b). Targeted knockdown of IL-13Rα2 in primary wtEGFR-positive GBM patient tumor, as validated by western blot analysis (Supplementary Fig. 7c), also did not affect cell proliferation (Supplementary Fig. 7d) nor cell migration (Supplementary Fig. 7e). Co-immunoprecipitation confirmed that IL-13Rα2 could interact with wtEGFR in Gli36.wtEGFR and tet-induced wtEGFR expressing U251-E6 cells (Fig. 6e), and in primary wtEGFR-positive GBM patient tumor (Fig. 6f). Despite the ability of IL-13Rα2 to interact with wtEGFR, no significant change in the levels of key MAPK, STAT3, and PI3K/AKT signaling proteins were observed when compared to wtEGFR (Supplementary Fig. 7f−h, respectively).

Next, we studied whether the difference between IL-13Rα2—mediated effects on cell proliferation in the presence of either WT or EGFRvIII may be attributed to differences in STAT3 activation. Targeted IL-13Rα2 knockdown showed only modestly reduced or no detectable change in STAT3 in wtEGFR-expressing cell line and primary glioma cultures derived from GBM patient tumors, respectively (Fig. 6g). In contrast, silencing of IL-13Rα2 in EGFRvIII-positive glioma cells and primary culture caused a significant reduction in STAT3 activation (Fig. 6h). TGF-β has been reported to increase glioma-initiating cell self-renewal via STAT3 activation^37^, and since IL-13 has been shown to induce TGF-β production via IL-13Rα2 in macrophages^38^, we therefore examined the expression levels of TGF-β in our isogenic glioma cell lines. The results demonstrate that TGF-β1 is markedly upregulated at the mRNA (Supplementary Fig. 4q) and at protein levels (Supplementary Fig. 4r) in cells co-expressing EGFRvIII and IL-13Rα2.

**Fig. 7:**
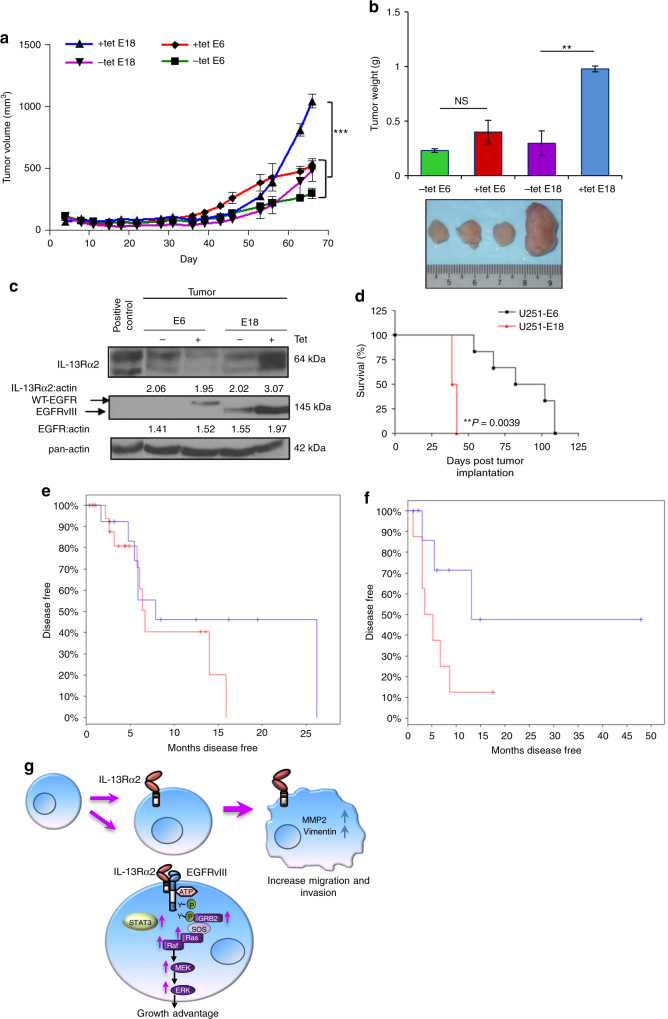
IL-13Rα2 mediate greater tumorigenic potential with EGFRvIII, and not WT EGFR. **a** Tumor volume **b** and tumor weight of tetracycline regulatable U251 gliomas (U251-E6 and U251-E18 was examined in vivo. Bars depict the mean values and error bars represent 95% confidence intervals. *P*-values were calculated using ANOVA with Tukey’s multiple comparison test *p < 0.05; **p < 0.01; ***p < 0.001. Photomicrographs of represented collected tumors are shown. **c** Immunoblot analysis of proteins from U251-E6 and -E18 tumor lysates in the presence or absence of tetracycline with the indicated antibodies. One representative tumor under each of the uninduced and induced conditions was shown. U251MG whole-cell lysate served as positive control for IL-13Rα2. **d** Kaplan−Meier survival curves of mice bearing U251-E6 and U251-18 tumors **p < 0.0039. Kaplan−Meier survival plots for patients expressing **e** high EGFR mRNA levels (excluding EGFRvIII) or **f** high EGFRvIII mRNA levels from TCGA database. High IL-13Rα2 expression (red) and low IL-13Rα2 expression (blue) were determined by aggregating all patients whose *z*-score normalized expression was above or below 0, respectively. **g** Schematic model showing signal transduction pathway co-induced by IL-13Rα2 and EGFRvIII. Overexpression of IL-13Rα2 in human gliomas increases cell migration and invasion through the activation of MMP-2, vimentin. Amplification of EGFRvIII promotes the co-interaction of both receptors mediating an increase in tyrosine kinase activities and a preferential activation of RAS-MEK-ERK and STAT3 pathways leading to aberrant cellular proliferation

The induction of cell proliferation in the presence of IL-13Rα2 and EGFRvIII, but not wtEGFR, was further investigated in vivo. The results showed that only tetracycline analogs fed mice, transplanted with U251-E18 cells expressing both IL-13Rα2 and mutant EGFR, had a significantly larger mean tumor volume (Fig. 7a) and tumor weight (Fig. 7b) compared to control groups or mice transplanted with wtEGFR, i.e., U251-E6 cells with or without doxycycline treatment. Immunoblotting analysis of the excised tumors in representative animals revealed a slight decrease in the level of IL-13Rα2 when wtEGFR proteins were induced, and a remarkable increase when mutant EGFR proteins were induced (Fig. 7c).

We also inoculated mice with U251-E6 and U251-E18 cells intracranially. The results showed that U251-E6 (wtEGFR and IL-13Rα2) mice remained symptom-free for significantly longer period when compared to U251-E18 (EGFRvIII and IL-13Rα2) bearing animals (median survival = 92 vs. 40.5 days, respectively (*p* = 0.0039) (Fig. 7d). More importantly, the co-expression of high levels of EGFR or EGFRvIII with IL-13Rα2 correlated with shorter patient survival by TGCA analysis (Fig. 7e, f, respectively). Taken together, these data demonstrated that IL-13Rα2 could collaborate with EGFRvIII, but not wtEGFR, at inducing cellular proliferation.

## Discussion

In brain tumors, high-grade gliomas typically consists of the central core and the infiltrative zone at the peripheral rim. The central core of the tumor divides rapidly while the peripheral rim of the tumor tends to invade the normal parenchymal, and paves the path to tumor recurrence. This phenomenon has been termed as “To Go or Grow” theory, where an inverse correlation of glioma cell motility and proliferation has been established^39^. In this study, we revealed a critical role for IL-13Rα2 in GBM tumor cell migration and growth; it can serve as a modulator between cellular migration and proliferation, depending in part on its ability to engage with other tyrosine kinase receptors crosstalk activities, thus, providing further support to the “To Go or Grow” theory during the progression of human gliomas (Fig. 7g).

A study that compared the expression of IL-13Rα2 from 8 publicly available gene expression data sets, including those derived from Phillips et al.^40^ and Verhaak et al.^41^ showed that IL-13Rα2 expression was positively associated with the mesenchymal signature genes and negatively associated with the proneural signature genes^21^. It may be worth noting that EGFRvIII expression is also significantly more frequent in glioma stem cells exhibiting mesenchymal-like expression signature compared to the proneural signature^42^. Report from other groups showed that GBM tumors positive for the ligands of IL-13Rα2 (i.e., CHI3L1/YKL40) and EGFRvIII are also clinically more aggressive compared to tumors lacking both molecular markers^6^, thus, suggesting a possible relationship between EGFRvIII/IL-13Rα2 and its ligand CHI3L1. Targeted silencing of CHI3L1 has been shown to overcome temozolomide-resistance in GBM^43^. IL-13Rα2 is also validated as the one of the 23 most upregulated genes, whose expression is predominantly restricted to human GBM tumors as confirmed by microarray transcription profiling, quantitative RT-PCR, and glycoproteomic analysis^44^. More recently, Xu et al.^45^ have further validated the microarray data and RNA-sequencing data of GBM downloaded from TCGA. Upon re-analysis, IL-13Rα2 is identified to be enriched in MAPK signaling pathway (*p* = 4.31 × 10^−4^) and the pathway associated with cancer (*p* = 3.48 × 10^−3^). Taken together, these findings are in agreement with our data that IL-13Rα2-positive cells exhibit elevated MAPK activities in a specific GBM cell population, i.e., the EGFRvIII+ cell types.

In our system, the overexpression of IL-13Rα2, in the absence of EGFRvIII, leads to GBM invasion. This is accompanied by the upregulation of epithelial mesenchymal transition (EMT) associated proteins, which is consistent with the finding that genes upregulated in the more invasive mesenchymal subtype of GBM overlaps significantly with the EMT signature^46^. IL-13Rα2 is also amongst the gene signatures that mediate breast cancer metastasis to the lung^47^ and to the brain^48^, thus, indicating the importance of IL-13Rα2 in promoting the invasion and migration potential of cancer cells. Ectopic expression of IL-13Rα2 proteins in the absence of EGFRvIII in Gli36 cells did not affect cell proliferation. These results are consistent with the findings of Kawakami et al.^49^ in that the overexpression of IL-13Rα2 proteins did not confer an added growth advantage in human breast and pancreatic cancer cell lines lacking the EGFRvIII receptors.

Previous studies have demonstrated that EGFRvIII is a gain-of-function mutation that confers greater tumorigenic potential as compared to the wild-type receptor in glioma cells and in transgenic mouse glioma models^50,51^. Despite that its constitutive activity is lower than that of the ligand-stimulated wtEGFR^4^, the mutant receptor is not downregulated unlike the wtEGFR following activation by ligand. This low level of sustained signaling through EGFRvIII is insufficient to account for all of its tumorigenic activities, which includes cell migration, cell survival, cell proliferation, and angiogenesis^4^. EGFRvIII has been shown to heterodimerize with wtEGFR leading to trans-phosphorylation of the wtEGFR^52^. Wild-type EGFR could also phosphorylate EGFRvIII in a STAT3/5 dependent manner, thus, promoting gliomagenesis^53^. In this study, the data demonstrates that the enhanced tyrosine kinase activities mediated by IL-13Rα2/EGFRvIII complex could lead to activation of oncogenic RAS, which subsequently activates the MEK/ERK signaling cascade under serum and serum-free culture condition. These findings are corroborated using primary EGFRvIII-positive GBM patient tumor, where IL-13Rα2 is silenced and in normal human astrocytes co-transfected with IL-13Rα2 and EGFRvIII. However, in these normal cells, we also observe a further increase in PI3K/Akt activation. We are uncertain what triggers this, but EGFRvIII has been shown to increase PI3K/Akt signalling in normal human astroyctes under DNA damage insults^54^.

A recent study demonstrated the heterodimerization of EGFRvIII with the cytokine oncostatin M receptor (OSMR), which is also a member of the interleukin-6 receptor family^55^. As a consequence, OSMR provided a positive feed-forward signal in the EGFRvIII-STAT3 pathway in driving gliomagenesis. Similar to OSMR, IL-13Rα2 is also a member of the type I cytokine receptor family and shares the common cytokine receptor homology module^12^, and is not known to harbor any STAT3 binding motif. However, the short intracellular domain of IL-13Rα2 has been shown to interact with the cytoplasmic domain of IL-4Rα chain that harbors the STAT docking sites^56^. Hence, it is likely that the interaction of IL-13Rα2 with EGFRvIII causes a conformational change in the receptor complex that favours increase in the recruitment and activation of STAT3. The major structural difference between EGFRvIII and wtEGFR lies in the gain-of-function mutation that arises from genomic deletion of exons 2–7 of EGFRvIII. A specific free cysteine residue present in EGFRvIII has been shown to be critical for the activation of EGFRvIII and for its ability to transactivate other receptor tyrosine kinases, such as c-Met^57^. This free cysteine residue is not present in the wild-type EGFR. Thus, it is likely that the absence of the free cysteine residue in wtEGFR has affected the ability of IL-13Rα2 to further activate the EGFRvIII-STAT3 axis. This is supported by our findings, whereby the STAT3 expression is significantly reduced in EGFRvIII-expressing cell line or patient-derived glioma culture, but to a much lesser extent in wtEGFR. Although it is tempting to speculate that the observed upregulation of TGF-β1 is due to heterodimerization of the IL-13Rα2 and EGFRvIII, which in turn leads to STAT3 activation and gliomagenesis, STAT3 is nevertheless a very complex signalling node with numerous pleiotropic upstream inputs. Thus, additional investigation will be required to further clarify the relationship between IL-13Rα2 and STAT3 signaling cascade.

In conclusion, we discovered a novel mechanism, whereby a non-tyrosine kinase decoy receptor (i.e., IL-13Rα2) is capable of regulating GBM tumorigenesis through the oncogenic signaling of a tyrosine kinase receptor (i.e., EGFRvIII). We demonstrated that the ectopic expression of the IL-13Rα2 receptor increases the invasive potential of GBM cells, but exerts no effect on cell proliferation. In contrast, IL-13Rα2 conferred enhanced proliferation through its association with EGFRvIII. The interaction leads to enhanced intrinsic phosphorylation of EGFR, which subsequently triggers the activation of RAS/RAF/MEK/ERK and STAT3 signaling cascades. Given that the co-expression of high levels of IL-13Rα2 and EGFRvIII/EGFR correlate with shorter patient survival by TGCA analysis, the activation of STAT3 by Y705 phosphorylation is linked with clinically more aggressive behavior in glioblastomas^58^. Our study suggests that targeting both receptors in conjunction with STAT3 signaling is a therapeutic approach to treat GBM patients.

## Methods

### Cell lines and cell proliferation assays

Human glioma cell lines Gli36, Gli36.EGFRvIII, Gli36.IL-13Rα2/EGFRvIII U251MG, U1242, U87MG, U87MG.EGFRvIII, U251-E18, U251-E6 and Mayo Clinic primary GBM xenograft cell lines were cultured in DMEM supplemented with 10% FBS (Gibco, CA, USA), 100 U/ml penicillin/streptomycin (Invitrogen Life Technologies, CA, USA), 2 mM L-glutamine (Sigma-Aldrich, MO, USA) and related selection antibiotic when required. Normal human astrocytes (NHA) (Lonza Bioscience, Basel, Switzerland) was cultured in Astrocyte Growth Medium (AGM) (Lonza Bioscience). Primary human brain tumors from National Neuroscience Institute (NNI, Singapore) were culture in ABM medium. All cells were maintained at 37^o^C in a 5% CO2-95% air atmosphere. The culture of primary glioma cells has been approved by the Centralized Institutional Review Board after obtaining patients informed consent. Cell proliferation was evaluated quantitatively at indicated time points after treatment or transfection with a metabolic indicator dye Alamar Blue (Biosource International) or with cell counting kit CCK-8 assay (Dojindo Laboratories, Kumamoto, Japan) according to manufacturer’s protocol.

### Plasmids and generation of stable clones

The full length (i.e., wild-type) IL-13Rα2 coding region was PCR amplified with forward (5′-GAGAAAGCTTATGGCTTTCGTTTGC-3′) and reverse (5′-GCCCTCTAGACTTATCGTCGTCATCCTTGTAATCTCATGTATCACAGAAAAATTC-3′) primers. The amplified sequence was subsequently cloned into pcDNA3.1 (Invitrogen Life Technologies). Positive clones were confirmed by both restriction enzyme digestion and DNA sequencing. Subsequently, the positive clone was transfected in Gli36 cells using standard Lipofectamine (Invitrogen Life Technologies) protocol. Single stable transfected clones were selected and maintained with 500 µg/ml of Geneticin (Sigma). Expression of IL-13Rα2 proteins was confirmed by flow cytometry and by immunoblotting.

### Co-immunoprecipitation

Glioma cells, either naive or after respective transfection, were cross-linked in Dithiobis (succinimidyl propionate; DSP) at room temperature (RT). After the reaction was stopped, cells were washed in cold PBS and centrifuged. Clarified lysates of 1 mg per sample were pre-cleared with protein G-sepharose beads and then incubated with anti-EGFR or anti-IL-13Rα2 antibody for immunoprecipitation. Samples were then analyzed by immunoblotting.

### Gene silencing, migration, and invasion assays

Glioma cells were transfected with either StealthTM RNAi duplexes (Invitrogen Life Technologies) or ON-TARGETplus SMARTPool RNAi duplexes (Dharmacon) targeting human IL-13Rα2 and CTRL-RNAi at a final concentration of 3–10 nM depending on the cell types. For migration or invasion assays, the cells were seeded on the top well of the trans-well migration (BD Biosciences) or Matrigel invasion chamber (BD Biosciences).

### Mice

All animal procedures were based on animal care guidelines approved by National Cancer Centre Animal Care and Use Committee.

### Immunohistochemistry

Primary antibodies used in IHC were as follows: IL-13Rα2 (AF146, R&D Systems), EGFR (MA5-13343, Thermo Fisher Scientific).

### Statistical analysis

The data are presented throughout this study as means ± standard error of the mean. The statistical significance was evaluated by an unpaired *t*-test, and *p* < 0.05 was considered significant. For in vivo studies, the mean values and error bars represent 95% confidence intervals. *P*-values were calculated using ANOVA with Tukey’s multiple comparison tests.

### Cell culture

Human glioma U87MG.EGFRvIII was engineered to express EGFRvIII proteins were cultured in G418 (0.5 mg/ml; Life Technologies, CA, USA) DMEM supplemented with 10% FBS (Gibco, CA, USA), 100 U/ml penicillin/streptomycin (Invitrogen Life Technologies, CA, USA), 2 mM l-glutamine (Sigma-Aldrich, MO, USA). The U251-E18 cells and U251-E6 cells expressed EGFRvIII and wt EGFR respectively in the presence of 1 µg/ml tetracycline (Tet) (Sigma-Aldrich). The inducibility of EGFRvIII (-E18) and wtEGFR (-E6) was confirmed by immunoblot analysis. The cells were maintained in standard tissue culture medium in the presence of 5 µg/ml Blasticidin and 250 µg/ml of Zeocin to prevent leakiness. All cells were maintained at 37 °C in a 5% CO2-95% air atmosphere. Human glioblastoma cell lines Gli36 (kind gift from A.T. Campagnoni, UCLA School of Medicine, CA, USA), U251MG (kind gift from D.F. Deen, Brain Tumor Research Centre, UCSF School of Medicine, CA, USA), U1242 (kind gift from Isa Hussaini, University of Virginia, Charlotesville, VA, USA), U87MG (American Type Culture Collection,Rockville, MD, USA) were cultured in 10% FBS containing DMEM supplemented with antibiotics and l-glutamine as described above. Human glioma cells Gli36 expressing a constitutively active variant of EGFRvIII (denoted as Gli36.EGFRvIII cells are kindly provided by Dr Esteves MS, University of Massachusetts, MA, USA) were cultured in standard tissue culture medium as described above, supplemented with 1 µg/ml puromycin (Sigma-Aldrich). All cell lines have been tested to be free of mycoplasma contamination by both PCR analysis and DNA florochrome staining method. Primary GBM xenograft cell lines GBM6, 10, 38, 46, and 59 were purchased from Mayo Clinic (Rochester, MN, USA) and maintained as subcutaneous xenografts as previously described. Normal human astrocytes (NHA) were purchased from Lonza Bioscience (Basel, Switzerland) and cultured as described in Astrocyte Growth Medium (AGM) (Lonza Bioscience). The culture of primary glioma cells has been approved by the Centralized Institutional Review Board after obtaining patients’ informed consent. Primary human brain tumors, which are pathologically confirmed as low and high-grade glioma, were obtained from National Neuroscience Institute (NNI, Singapore). The collected tissues were rinsed three times in ice-cold Hank’s Buffered Salt Solution (HBSS) (Invitrogen Life Technologies) without calcium and magnesium to remove excess blood and sliced finely to yield ~1-mm^3^ fragment. The tumor fragments were digested in 0.25% trypsin-EDTA (Invitrogen Life Technologies) at 37 °C for 30 min with constant stirring, and subsequently resuspended in equal volume of complete Astrocyte Basal Medium (ABM) media (Lonza). The cell suspension was then centrifuged at 1000 r.p.m. for 5 min and the cell pellet was resuspended in fresh growth medium. The resulting cell suspension was strained through a 70 µm cell strainer (BD Biosciences, NJ, USA), rinsed once with phosphate buffered saline (PBS) prior to culturing in ABM medium at 37 °C in a humidified incubator with 5% CO_2_.

### Plasmids constructs and stable clones

The pcDNA-IL-13Rα2 plasmid was transfected in Gli36 cells using standard Lipofectamine (Invitrogen Life Technologies) protocol. Single stable transfected clones were selected and maintained with 500 µg/ml of Geneticin (Sigma). Expression of IL-13Rα2 proteins was confirmed by flow cytometry (FACSCanto II; BD Biosciences) using anti-IL-13Rα2 antibodies (R&D systems, MN, USA) and by immunoblotting.

### Transfection

The entire cytoplasmic region (amino acids 364–380) of the IL-13Ra2 was replaced with an alanine residue by using inverse-PCR method with a forward primer 5′-GCTTGAAGACTTTCCATATCAAGAGAC-3′(stop codon is underlined) and a reverse primer 5′-CAAAAGCAGACCGGTTACAAATATAACT-3′. This was constructed in pIRESNeo-IL-13Rα2 plasmid. Subsequently, Gli36.EGFRvIII were transfected with either pIRESNeo vector, wild-type IL-13Rα2 or IL-13Rα2 ΔCyt tail mutant, and the corresponding Gli36.IL-13Rα2 cells were transfected with full length EGFRvIII, EGFRvIII kinase dead (DK), EGFRvIII site mutation (DY3) using standard Lipofectamine/PLUS (Invitrogen Life Technologies) following manufacturer’s instructions. After 48 h, cells were collected for the co-immunoprecipitation assay or cell proliferation assays. NHA were transfected with either pIRESneo vector, full length IL-13Rα2, pLRNL.EGFRvIII or both receptors using jetPRIME transfection reagent (Polyplus Transfection, NY, USA) following manufacturer’s instructions in serum-free culture conditions. After 48 h, cells were harvested for immunoblotting.

### Antibodies

Western blotting was performed using the following primary antibodies: anti-IL-13Rα2 (R&D systems, AF146; 1/1000), anti-EGFR clone 12 (Thermo Scientific/NeoMarker, NH, USA, MS-400-P; 1/1000), anti-EGFR (Cell Signaling Technology, MA, USA, #4267; 1/1000), anti-MMP2 (Santa Cruz Biotechnology, sc-10736; 1/1000), anti-vimentin (Epitomics, Burlingame, CA, USA, 2862-1; 1:1000), anti-Grb2 (BD Transduction Lab, NJ, USA, G16720; 1/1000), anti-phosphotyrosine (clone 4G10) (Upstate/Millipore, MA, USA, 05-321; 1/1000), anti-phosphotyrosine (p-Tyr-102) (Cell Signaling Technology, #9416; 1/1000), anti-pan Ras (Calbiochem, MA, USA, #OP-40; 1/1000), anti-C-Raf (BD Transduction Lab, R19120; 1/1000), anti-phospho-C-Raf (Ser338) (Cell Signaling Technology, #9427; 1/1000), anti-MEK1/2 (Cell Signaling Technology, #9122; 1/1000), anti-phospho-MEK1/2 (Ser217/221) (Cell Signaling Technology, #9121; 1/1000), anti-p42/44 MAPK (ERK1/2) (Cell Signaling Technology, #4695; 1/1000), anti-phospho-p42/44 MAPK (ERK1/2) (Thr-202/Tyr-204) (Cell Signaling Technology, #9101; 1/1000), anti-PTEN (Cell Signaling Technology, #9559; 1/1000), anti-PI3K p85α (Cell Signaling Technology, #4292; 1/1000), anti-phospho-PI3K p85α (Y458) (Cell Signaling Technology, #4228; 1/1000), anti-AKT (Cell Signaling Technology, #4691; 1/1000), anti-phospho-AKT (Ser-473) (Cell Signaling Technology, #9271; 1/1000), anti-STAT3 (Cell Signaling Technology, #9139; 1/1000), anti-phosho-STAT3 (Ser-727) (Cell Signaling Technology, #9136; 1/1000), anti-phospho-STAT3 (Tyr705) (Cell Signaling Technology, #9131; 1/1000), anti-TGFβ (Cell Signaling Technology, #3711; 1/1000), anti-Hsp70 (System Biosciences, CA, USA, H53220; 1/1000), anti-pan-actin (Thermo Scientific/Neomarker, MS-1295-P; 1/ 20,000), anti-β-tubulin (BD Biosciences, 556321; 1/5000) and anti-tubulin (Santa Cruz Biotechnology, sc-5286; 1/10,000).

### Cell cycle analysis

Human glioma cells were fixed with 500 μl of ice-cold 70% ethanol overnight at 4 °C, rinsed in PBS supplemented with 200 μl of RNase A (2 mg/ml; Sigma-Aldrich) and 200 μl of PI (100 μg/ml; Sigma-Aldrich). The samples were kept on ice for at least 1 h in the dark prior to FACS analysis.

### Cell proliferation assays

Serum-free and serum-containing cultures were plated in DMEM supplemented with B27 (Invitrogen Life Technologies) and 10% FBS/DMEM. Cell proliferation assay was determined by cell counting kit CCK-8 assay (Dojindo Laboratories, Kumamoto, Japan) at optical density 450 nm using the Victor spectrophotometer (PerkinElmer Life Sciences, MA, USA). For the Tet-inducible growth kinetic assay, U251-E6 and U251-E18 cells were seeded in a 96-well plate at a density of at 5000 cells per well. After 24 h, cells were either assayed at Day 1 (without Tet) for cell viability or cultured in the presence or absence of 1 µg/mL Tet (Sigma-Aldrich) diluted in serum-free DMEM. Subsequently, the cell viability of these cells was assayed at Day 2 and Day 4. For AG1478 treatment, glioma cell lines and primary glioma cells were cultured under serum-free condition. The cells were incubated, with or without AG1478 (10 µM, Sigma-Aldrich) for 24 h prior to CCK-8 assay or western blot analysis.

### Rembrandt dataset (publicly available data set)

To correlate IL-13Rα2 expression with survival, the National Cancer Institute’s Repository for Molecular Brain Neoplasia Data (REMBRANDT) database was used. To correlate the co-expression of IL-13Rα2 and EGFR with survival, glioma patient cohorts with twofold higher expression of EGFR mRNA was first selected. These groups of patients were further stratified into groups having higher level of IL-13Rα2 (twofold upregulation), intermediate or lower level of IL-13Rα2 (twofold downregulation). The correlation between EGFR and IL-13Rα2 expression levels in all glioma patients selected in this database was further examined using chi-square distribution (*p* = 0.0399; Fisher’s exact test 0.0328).

### Colony formation in semi-solid medium

In brief, cells at a density of (3.5 × 10^4^/well) were resuspended in 1X Iscove’s modified Dulbecco’s medium (Invitrogen Life Technologies) with 10% FCS in 0.35% (w/v) top Noble Agar (w/v) (Sigma-Aldrich) with a base bottom agar of 0.7% agar. After 10 days, colonies were stained with 0.005% crystal violet (Sigma-Aldrich), colonies were counted in five randomly selected field at ×10 magnification. Results were obtained with two independent experiments, each in quadruplicates.

### Gene silencing, migration and invasion assays

Gene knockdown by siRNA was accomplished by transfecting Stealth RNAi duplexes (Invitrogen Life Technologies) pre-designed for human IL-13Rα2 or ON-TARGETplus SMARTPool human IL-13Rα2 siRNA and non-targeting pool siRNA (Dharmacon, CO, USA) at a final concentration of 3-10 nM using Lipofectamine RNAiMax (Invitrogen Life Technologies) according to manufacturer’s instruction. Proliferation assay using CCK-8 was performed at the indicated time points. For migration or invasion assays, the transfected cells were seeded to the top well of the 8 μm migration (BD Biosciences) or Matrigel invasion chamber (BD Biosciences). Migrated cells were fixed with 4% paraformaldehyde (PFA) after 8 h and mounted in mounting medium containing propidium iodide (PI; 100 µg/ml) and RNase (2 mg/ml; Sigma-Aldrich). YKL-40 i.e., CHI3L1 was from R&D Systems and IL-13 was from Peprotech. The percentage of migrated cells was subsequently quantified by counting the number of PI-stained nuclei cells in the bottom side of the membrane from at least five random fields at ×200 magnification.

### In vivo mouse studies

All animal experiments were performed according to the guidelines and protocols approved by the SingHealth Institutional Animal Care and Use Committee, Singapore. Details of the different animal models are as follow:

### Subcutaneous mouse xenograft

Six to eight-week-old female immunodeficient NOD/SCID mice (Animal Resource Centre, Canning Vale, Western Australia) were injected subcutaneously with U251-E6 and U251-E18 cells (5 × 10^6^ cell each, *n* = 4 per group) resuspended in a 1:1 mixture of PBS and Matrigel (BD Bioscience) in the right and left flanks, respectively. The tumors were allowed to grow for 2 months, monitored closely by twice weekly measurements, and were finally weighed at sampling. For doxycycline-treated mice, doxycycline (0.2 mg/ml) was added to the drinking water a week prior to the injection of cells. The tumor volume was measured and calculated according to the formula volume = 0.52 × length × width^2^. At the end of the experimental period, all animals were killed and tumor nodules were weighed before collect. Tumor nodules from representative animals were collected at day 66, homogenized in presence of chilled homogenization buffer (1 M Sucrose, 0.5 M EDTA pH 8.0, 1 M Tris-HCl pH 7.2, 100 mM PMSF) containing Halt protease and phosphatase inhibitors (Roche, Basel, Switzerland) for protein analysis. Homogenized samples were clarified by centrifugation for 10 min at 4 °C, and concentrated using Vivaspin-2 centrifugal concentrators (Sartorius Stedim, Göttingen, Germany) for 30 min. After normalizing for protein concentrations, immunoblot procedures were performed with the relevant antibodies.

### Intracranial U251-E6 and E18 mouse xenograft

U251MG derived cells (2 × 10^6^) cells were suspended in Matrigel/PBS (5 µl) and injected into the right corpus striatum of the brains of 6–8-week-old nude mice using a stereotactic frame. Animals were monitored and killed when neurological signs appeared. Kaplan−Meier survival analyses and statistical analyses were performed using GraphPad Prism software (version 3.03).

### Intracranial glioma mouse xenograft

Gli36.IL-13Rα2 (2 × 10^5^ cells) was stably transduced with lentivirus pWPT-GFP, followed by implantation into the right hemisphere (Bregma (0, 0) lateral 2 mm and depth 2.5 mm) of 6−8-week-old female immunodeficient Balb-C nu/nu mice (Animal Resource Centre). Glioma-bearing mice were killed 21 days post-tumor implantation and immediately perfused through the heart with ice-cold PBS. Tumors were collected and fixed in 4% PFA and 30% sucrose. Cryosections (10 µm) were analyzed histologically using standard H&E staining. For immunofluorescence staining, cryosections were washed in PBS followed by permeabilization with 0.2% triton X-100 for 10 min room temperature (RT). Blocking of sections was done with 5% BSA and 0.2% triton X-100 (in PBS) for 1 h RT. Sections were then incubated with goat anti-human IL-13Rα2 (R&D Systems; 15 µg/ml), anti-MMP2 (Santa Cruz Biotechnology; 1/100), and anti-vimentin (Epitomics; 1/1000) for 2 h RT. After several washes, slides were incubated with rabbit anti-goat AlexaFluor 594 secondary antibody for 1 h RT. Nuclei were counterstained with DAPI (1 µg/ml) for 5 min RT and then slides were mounted using Slow Fade (Invitrogen Life Technologies). Slides were visualized using confocal microscopy (LSM 510 Meta; Carl Zeiss, Göttingen, Germany) and images were obtained using either a ×40/0.75 numerical aperture (N.A) Plan-Neofluar or 20x/0.75 N.A objective (Carl Zeiss). Similar experiments were performed for Kaplan−Meir survival analysis using Gli36, Gli36.IL-13Rα2, Gli36.EGFRvIII, and Gli36.IL-13Rα2/EGFRvIII.

### Co-localization studies

For co-localization studies, naive cells or cells transfected with full length EGFRvIII or variants were seeded at 8 × 10^4^ cells in 12-well dish. Following day, cells were fixed in 4% PFA for 10 min RT and permeabilized with 0.1% Triton X-100 for 5 min RT. Cells were first blocked with 5% rabbit serum containing 0.3 M glycine (Invitrogen Life technologies) and PBS with 0.1% tween 20 for 1 h RT. Then, they were incubated with anti-human IL-13Rα2 (R&D Systems; 15 µg/ml) at 4 ^o^C overnight. After several washes, cells were incubated with rabbit anti-goat AlexaFluor 594 secondary antibody (Invitrogen Life Technologies) for 1 h RT. Following few rinses in PBS with 0.1% tween 20, cells were blocked second time with 5% goat serum containing 0.3 M glycine and PBS with 0.1% tween 20 for 1 h RT. Then cells were incubated with mouse anti-human EGFR (DakoCytomation; 1/75) for 2 h RT. After washes in 0.1% PBT, cells were incubated with goat anti-mouse AlexaFluor 488 for 1 h RT. Nuclei were counterstained with DAPI (1 µg/ml) for 5 min RT and then coverslips were mounted using Slow Fade (Invitrogen Life Technologies).

### Immunohistochemistry staining

Brain tissue was fixation with 4% paraformaldehyde (PFA), process and embedded in paraffin. Section slide were subjected to antigen retrieval with sodium citrate buffer, pH 6.0. Endogenous peroxidase was quenched with 0.3% H_2_O_2_ for 20 min and then blocked with 5% BSA at RT. Primary antibodies used in IHC were as follows: IL-13Ra2 (AF146, R&D Systems), EGFR (MA5-13343, Thermo Fisher Scientific). After washing, sections were incubated with either respective secondary antibody before DAB chromogenic detection. The sections were then counterstained with Hematoxylin prior to mounting and visualization.

### Immunoblot analysis

Cells were lysed in lysis buffer (50 mM Tris, 150 mM NaCl, 1% triton X-100) supplemented with fresh protease inhibitor cocktail (Roche), and phosphatase inhibitor (Sigma-Aldrich). For brain tissue specimens, the samples were homogenized with protein extraction buffer (1% Triton X-100, 300 mM NaCl, 2 mM EDTA, 400 µM Na_3_VO_4_, 1% NP-40) supplemented with fresh protease and phosphatase inhibitors. Cell extracts were incubated on ice for 10 min and clarified by centrifugation at 10,000 r.p.m. for 10 min at 4 ^o^C. Protein concentration was determined by Bradford protein assay (Bio-Rad Laboratories, CA, USA). Briefly 40–100 µg of proteins was resolved by 8–15% SDS-PAGE and electroblotted to polyvinylidene fluoride (PVDF) membrane (Trans-Blot Transfer medium; Bio-Rad Laboratories). Membranes were immunoblotted with indicated primary antibodies diluted in blocking buffer. The blocking buffer consists of either PBS with 5% bovine serum albumin (Thermo Scientific) or 5% non-fat milk power in PBS consisting of 0.1% tween 20 (Sigma). The rinsed membrane was subsequently incubated with secondary antibodies including goat anti-rabbit, goat anti-mouse immunoglobulin (DakoCytomation; 1/20,000) or rabbit anti-goat immunoglobulin (Santa Cruz Biotechnology; 1/10,000) for 1 h RT. Immunoreactivity was detected with Western Lightning chemiluminescent kit (PerkinElmer). The band density of specific proteins was quantified using either MetaVue (Ver. 6.1) (Molecular Devices Corp, CA, USA) or ImageJ (NIH, MD, USA) software. For all immunoblots, pan-actin served as internal loading controls. The values derived are from either analyzing a single experiment normalizing each of the signaling proteins against the internal control, or as a ratio of phosphorylated form over the total protein. Each of these was performed at least two independent times.

### Co-Immunoprecipitation (co-IP)

Human glioma cells were seeded at 2 × 10^6^ cells per 100 mm dish in complete medium. Exponentially growing cells were washed in PBS before crosslinking in 2 mM DSP diluted in PBS for 1 h RT. Crosslinking reaction was stopped with 20 mM Tris, pH 7.5 for 15 min at RT. Subsequently cells were washed again in cold PBS, gently scrapped off and centrifuged at 1000 r.p.m. for 5 min. Cell pellet was then lysed with NP-40 lysis buffer (50 mM Tris-HCl pH 8.0, 150 mM NaCl, 1% NP-40) supplemented with protease and phosphatase inhibitors (Roche).

Lysates were homogenized through 23.5 gauge needles, incubated on ice for 15 min and then clarified by centrifugation at 13,400 r.p.m. for 15 min at 4 °C. Clarified lysates of 1 mg per sample were pre-cleared with non-specific IgG antibody, following by adding protein G Agarose beads (Millipore). The pre-cleared lysates were subsequently incubated with 2 µg of specific antibody (anti-IL-13Rα2, anti-EGFR or anti-Grb2) for 4 h at 4 °C. Thereafter, protein G beads were added to the samples and the reaction mixture was incubated overnight at 4 °C. Following day, after the beads were washed three times in ice-cold NP-40 buffer, 2X laemmli buffer was added and heated at 95 °C for 10 min. Both IP and total input samples were loaded unto 8% SDS-PAGE and analyzed by immunoblotting.

### Proximity ligation assay

Gli36.EGFRvIII cells were transfected with pIRESneo2 encoding either wild-type IL-13Rα2, IL-13Rα2 cytoplasmic deleted mutant or empty vector alone. Twenty four hours post transfection, the cells were seeded on an eight-chamber slide (Thermo Scientific), and incubated for a further 24 h prior to fixation and permeabilization in 0.1% Triton X-100 in PBS. Non-specific binding was blocked by incubation with Blocking Solution (Duolink, Sigma-Aldrich) for 1 h at RT. Rabbit monoclonal antibody targeting targeting EGFR (Clone D38B1, Cell Signaling Technology) was incubated overnight at 4 °C together with goat antibody targeting IL13Rα2 (AF146, R&D Systems). PLA minus probes against rabbit and plus against goat from (Duolink, Sigma-Aldrich) were used in conjunction with Duolink In Situ PLA Red Kit (Duolink, Sigma-Aldrich) according to the manufacturer’s description. Negative controls were carried out in the absence of primary or secondary antibody while stable clones Gli36.IL-13Rα2/EGFRvIII serve as positive controls. The PLA signals (red), indicative of sites of protein-protein interaction, were observed and the images were captured using under the fluorescence microscope (Olympus, Tokyo, Japan) at ×400 magnification. Similar experiments were performed with Gli36.IL-13Rα2 cells transfected with full length EGFRvIII, DK and DY3 mutants.

### Ras activation assay

Ras is cycling between an inactive GDP-bound form and an active GTP-bound form. In its GTP-bound active state, Ras binds specifically to the Ras-binding domain (RBD) of Raf-1 to control downstream signaling cascades. GTP-Bound Active RAS pull-down assay was performed using the Raf-1 RBD agarose beads (Cat#STA-400, Cell Biolabs, CA, USA) which it interacts and precipitates GTP-bound Ras from cell lysate and pull-down only the active Ras. Cells were serum starved overnight before collected for Ras activation assay. Fresh lysates from each cell lines were incubated with 30 µl of Raf-1 RBD agarose beads for 3 h at 4 °C. GTPγS and GDP were loaded to serve as positive and negative control, respectively. The precipitated GTP-Ras was eluted in 2x SDS sample buffer and visualized by immunoblotting with anti-pan-Ras antibodies. The levels of Ras activation are then expressed as a ratio of Ras-GTP to the total Ras/actin levels in the same lysates. Ras activation is also measured using ELISA kit (Cat#STA-440, Cell Biolabs) in accordance to the manufacturer’s instruction. Absorbance of each microwell was read on a spectrophotometer using 450 nm as the primary wave length.

### RNA isolation and qRT-PCR (Quantitative reverse transcription PCR)

Total RNA was isolated using TRIzol Reagent according to the manufacturer’s instructions (QIAGEN, CA, USA). Total RNA from samples was reverse transcribed into cDNA using RevertAid First Strand cDNA Synthesis Kit (Thermo Scientific). Real-time PCR was performed on CFX96 Touch (Bio-Rad Laboratories) using QuantiTect SYBR Green PCR Kit (QIAGEN). Amplification was done under following conditions: 95 °C for 15 min; followed by 40 cycles of 94 °C, 15 s; 55 °C, 30 s, and 72 °C, 30 s. GAPDH was use as endogenous control. The 2^−ΔΔCt^ method was used to calculate fold changes in gene expression. Each sample was run in triplicates and at least three experiments were analyzed. Primer sequences used are: GAPDH forward sequence 5′-GAAGGTGAAGGTCGGAGTCA-3′; reverse sequence 5′-TTGAGGTCAATGAAGGGGTC-3′, TGFβ forward sequence 5′-CACTCTCAAACCTTTACGAGACC-3′; reverse sequence 5′-CGTTGCTAGGGGCGAAGATG-3′.

### Data availability

All relevant data are available from the corresponding authors upon request.

## Electronic supplementary material

Supplementary Information
