# Supplementary material for: Interleukin-13 receptor alpha 2 cooperates with EGFRvIII signaling to promote glioblastoma multiforme
Source: Nat Commun. 2017 Dec 4;8:1913. doi: 10.1038/s41467-017-01392-9 (PMC5715073; doi:10.1038/s41467-017-01392-9)
Supplement: Supplementary file 1 — Supplementary Information [file 41467_2017_1392_MOESM1_ESM.pdf]

Supplementray Methods

Cell Culture

Human glioma U87MG.EGFRvIII was engineered to express EGFRvIII proteins were cultured in G418 (0.5mg/ml; Life Technologies, CA, USA) DMEM supplemented with 10% FBS (Gibco, CA, USA), 100 U/ml penicillin/streptomycin (Invitrogen Life Technologies, CA, USA), 2 mM L-glutamine (Sigma-Aldrich, MO, USA). The U251-E18 cells and U251-E6 cells expressed EGFRvIII and wt EGFR respectively in the presence of 1 µg/ml tetracycline (Tet) (Sigma-Aldrich)[1]. The inducibility of EGFRvIII (-E18) and wtEGFR (-E6) was confirmed by immunoblot analysis. The cells were maintained in standard tissue culture medium in the presence of 5 µg/ml Blasticidin and 250 µg/ml of Zeocin to prevent leakiness. All cells were maintained at 37ºC in a 5% CO_2-95% air atmosphere. Human glioblastoma cell lines Gli36 (kind gift from A.T. Campagnoni, UCLA School of Medicine, CA, USA), U251MG (kind gift from D.F. Deen, Brain Tumor Research Centre, UCSF School of Medicine, CA, USA), U1242 (kind gift from Isa Hussaini, University of Virginia, Charlotesville, VA, USA), U87MG (American Type Culture Collection,Rockville, MD, USA) were cultured in 10% FBS containing DMEM supplemented with antibiotics and L-glutamine as described above. Human glioma cells Gli36 expressing a constitutively active variant of EGFRvIII (denoted as Gli36.EGFRvIII cells are kindly provided by Dr Esteves MS, University of Massachusetts, MA, USA) were cultured in standard tissue culture medium as described above, supplemented with 1 µg/ml puromycin (Sigma-Aldrich). All cell lines have been tested to be free of mycoplasma contamination by both PCR analysis and DNA florochrome staining method. Primary GBM xenograft cell lines GBM6, 10, 38, 46 and 59 were purchased from Mayo Clinic (Rochester, MN, USA) and maintained as subcutaneous xenografts as previously described[2]. Normal human astrocytes (NHA) were purchased from Lonza Bioscience (Basel, Switzerland) and cultured as described in Astrocyte Growth Medium (AGM) (Lonza Bioscience). The culture of primary glioma cells has been approved by the Centralized Institutional Review Board after obtaining patients' informed consent. Primary human brain tumors, which are pathologically confirmed as low and high-grade glioma, were obtained from National Neuroscience Institute (NNI, Singapore). The harvested tissues were rinsed three times in ice-cold Hank's Buffered Salt Solution (HBSS) (Invitrogen Life Technologies) without calcium and magnesium to remove excess blood and sliced finely to yield approximately 1-mm^3 fragment. The tumor fragments were digested in 0.25% trypsin-EDTA (Invitrogen Life Technologies) at 37°C for 30 min with constant stirring, and subsequently resuspended in equal volume of complete Astrocyte Basal Medium (ABM) media (Lonza). The cell suspension was then centrifuged at 1000 rpm for 5 min and the cell pellet was resuspended in fresh growth medium. The resulting cell suspension was strained through a 70 µm cell strainer (BD Biosciences, NJ, USA), rinsed once with phosphate buffered saline (PBS) prior to culturing in ABM medium at 37°C in a humidified incubator with 5% CO_2.

Plasmids Constructs and stable clones

The pcDNA-IL-13Rα2 plasmid was transfected in Gli36 cells using standard Lipofectamine (Invitrogen Life Technologies) protocol. Single stable transfected clones were selected and maintained with 500 µg/ml of Geneticin (Sigma). Expression of IL-13Rα2 proteins was confirmed by flow cytometry (FACSCanto II; BD Biosciences) using anti-IL-13Rα2 antibodies (R&D systems, MN, USA) and by immunoblotting.

Transfection

The entire cytoplasmic region (amino acids 364-380) of the IL-13Ra2 was replaced with an alanine residue by using inverse-PCR method with a forward primer 5'-GCT<u>TGA</u>AGACTTTCCATATCAAGAGAC-3'(stop codon is underlined) and a reverse primer 5'-CAAAAGCAGACCGGTTACAAATATAACT-3'. This was constructed in pIRESNeo-IL-13Rα2 plasmid. Subsequently, Gli36.EGFRvIII were transfected with either pIRESNeo vector, wild-type IL-13Rα2 or IL-13Rα2 ΔCyt tail mutant, and the corresponding Gli36.IL-13Rα2 cells were transfected with full length EGFRvIII, EGFRvIII kinase dead (DK), EGFRvIII site mutation (DY3) using standard Lipofectamine/PLUS (Invitrogen Life Technologies) following manufacturer's instructions. After 48 h, cells were harvested for the co-immunoprecipitation assay or cell proliferation assays. NHA were transfected with either pIRESneo vector, full length IL-13Rα2, pLRNL.EGFRvIII or both receptors using jetPRIME transfection reagent (Polyplus Transfection, NY, USA) following manufacturer's instructions in serum-free culture conditions. After 48 h, cells were harvested for immunoblotting.

Antibodies Western blotting was performed using the following primary antibodies: anti-IL-13Rα2 (R&D systems, AF146; 1/1000), anti-EGFR clone 12 (Thermo Scientific/NeoMarker, NH, USA, MS-400-P; 1/1000), anti-EGFR (Cell Signaling Technology, MA, USA, #4267; 1/1000), anti-MMP-2 (Santa Cruz Biotechnology, sc-10736; 1/1000), anti-vimentin (Epitomics, Burlingame, CA, USA, 2862-1; 1:1000), anti-Grb2 (BD Transduction Lab, NJ, USA, G16720; 1/1000), anti-phosphotyrosine (clone 4G10) (Upstate/Millipore, MA, USA, 05-321; 1/1000), anti-phospho-Tyrosine (p-Tyr-102) (Cell Signaling Technology, #9416; 1/1000), anti-pan Ras (Calbiochem, MA, USA, #OP-40; 1/1000), anti-C-Raf (BD Transduction Lab, R19120; 1/1000), anti-phospho-C-Raf (Ser-338) (Cell Signaling Technology, #9427; 1/1000), anti-MEK1/2 (Cell Signaling Technology, #9122; 1/1000), anti-phospho-MEK1/2 (Ser217/221) (Cell Signaling Technology, #9121; 1/1000), anti-p42/44 MAPK (ERK1/2) (Cell Signaling Technology, #4695; 1/1000), anti-phospho-p42/44 MAPK (ERK1/2) (Thr-202/Tyr-204) (Cell Signaling Technology, #9101; 1/1000), anti-PTEN (Cell Signaling Technology, #9559; 1/1000), anti-PI3K p85α (Cell Signaling Technology, #4292; 1/1000), anti-phospho-PI3K p85α (Y458) (Cell Signaling Technology, #4228; 1/1000), anti-AKT (Cell Signaling Technology, #4691; 1/1000), anti-phospho-AKT (Ser-473) (Cell Signaling Technology, #9271; 1/1000), anti-STAT3 (Cell Signaling Technology, #9139; 1/1000), anti-phosho-STAT3 (Ser-727) (Cell Signaling Technology, #9136; 1/1000), anti-phospho-STAT3 (Tyr-705) (Cell Signaling Technology, #9131; 1/1000), anti-TGFβ (Cell Signaling Technology, #3711; 1/1000), anti-Hsp70 (System Biosciences, CA, USA, H53220; 1/1000), anti-pan actin (Thermo Scientific/Neomarker, MS-1295-P; 1/ 20 000), anti-β-tubulin (BD Biosciences, 556321; 1/5000) and anti-tubulin (Santa Cruz Biotechnology, sc-5286; 1/10 000).

Cell Cycle Analysis
Human glioma cells were fixed with 500 µl of ice cold 70% ethanol overnight at 4 °C, rinsed in PBS supplemented with 200 µl of RNase A (2 mg/ml; Sigma-Aldrich) and 200 µl of PI (100 µg/ml; Sigma-Aldrich). The samples were kept on ice for at least 1 h in the dark prior to FACS analysis.

Cell Proliferation Assays
Serum-free and serum-containing cultures were plated in DMEM supplemented with B27 (Invitrogen Life Technologies) and 10% FBS/DMEM. Cell proliferation assay was determined by cell counting kit CCK-8 assay (Dojindo Laboratories, Kumamoto, Japan) at optical density 450 nm using the Victor spectrophotometer (PerkinElmer Life Sciences, MA, USA). For the Tet-inducible growth kinetic assay, U251-E6 and U251-E18 cells were seeded in a 96-well plate at a density of at 5000 cells per well. After 24 h, cells were either assayed at Day 1 (without Tet) for cell viability or cultured in the presence or absence of 1 µg/mL Tet (Sigma-Aldrich) diluted in serum-free DMEM. Subsequently, the cell viability of these cells was assayed at Day 2 and Day 4. For AG1478 treatment, glioma cell lines and primary glioma cells were cultured under serum free condition. The cells were incubated, with or without AG1478 (10 µM, Sigma-Aldrich) for 24 h prior to CCK-8 assay or western blot analysis.

Rembrandt Dataset (publicly available dataset)
To correlate IL-13Rα2 expression with survival, the National Cancer Institute's Repository for Molecular Brain Neoplasia Data (REMBRANDT) database was used. To correlate the co-expression of IL-13Rα2 and EGFR with survival, glioma patient cohorts with 2-fold higher expression of EGFR mRNA was first selected. These groups of patients were further stratified into groups having higher level of IL-13Rα2 (2-fold upregulation), intermediate or lower level of IL-13Rα2 (2-fold downregulation). The correlation between EGFR and IL-13Rα2 expression levels in all glioma patients selected in this database was further examined using chi-square distribution (p = 0.0399; Fisher's exact test 0.0328).

Colony formation in semi-solid medium
In brief, cells at a density of (3.5 x 10^4/well) were resuspended in 1x Iscove's modified Dulbecco's medium (Invitrogen Life Technologies) with 10% FCS in 0.35 % (w/v) top Noble Agar (w/v) (Sigma-Aldrich) with a base bottom agar of 0.7 % agar. After 10 days, colonies were stained with 0.005% crystal violet (Sigma-Aldrich), colonies were counted in five randomly selected field at x 10 magnification. Results were obtained with two independent experiments, each in quadruplicates.

Gene silencing, migration and invasion assays

Gene knockdown by siRNA was accomplished by transfecting Stealth RNAi™ duplexes (Invitrogen Life Technologies) pre-designed for human IL-13Rα2 or ON-TARGETplus SMARTPool human IL-13Rα2 siRNA and non-targeting pool siRNA (Dharmacon, CO, USA) at a final concentration of 10 nM using Lipofectamine RNAiMax (Invitrogen Life Technologies) according to manufacturer's instruction. For U251MG and Gli36 cells, the optimum RNAi used was 3 nM of RNAi duplex was used. Proliferation assay using CCK-8 was performed at the indicated time points. For migration or invasion assays, the transfected cells were seeded to the top well of the 8 µm migration (BD Biosciences) or Matrigel invasion chamber (BD Biosciences). Migrated cells were fixed with 4% paraformaldehyde (PFA) after 8 h and mounted in mounting medium containing propidium iodide (PI; 100 µg/ml) and RNase (2 mg/ml; Sigma-Aldrich). YKL-40 i.e. CHI3L1 was from R&D Systems and IL-13 was from Peprotech. The percentage of migrated cells was subsequently quantified by counting the number of PI-stained nuclei cells in the bottom side of the membrane from at least 5 random fields at 200x magnification.

***In Vivo* Mouse Studies**

All animal experiments were performed according to the guidelines and protocols approved by the SingHealth Institutional Animal Care and Use Committee, Singapore. Details of the different animal models are as follow:

Subcutaneous mouse xenograft

Six to eight week old female immunodeficient NOD/SCID mice (Animal Resource Centre, Canning Vale, Western Australia) were injected subcutaneously with U251-E6 and U251-E18 cells (5 x 10^6 cell each, n = 4 per group) resuspended in a 1:1 mixture of PBS and Matrigel (BD Bioscience) in the right and left flanks respectively. The tumors were allowed to grow for two months, monitored closely by twice weekly measurements, and were finally weighed at sampling. For doxycycline-treated mice, doxycycline (0.2 mg/ml) was added to the drinking water a week prior to the injection of cells. The tumor volume was measured and calculated according to the formula $volume = 0.52 \times length \times width^2$. At the end of the experimental period, all animals were sacrificed and tumor nodules were weighed before harvest. Tumor nodules from representative animals were harvested at day 66, homogenized in presence of chilled homogenization buffer [1M Sucrose, 0.5M EDTA pH8.0, 1M Tris-HCL pH7.2, 100mM PMSF] containing Halt protease and phosphatase inhibitors (Roche, Basel, Switzerland) for protein analysis. Homogenized samples were clarified by centrifugation for 10 min at 4°C, and concentrated using Vivaspin-2 centrifugal concentrators (Sartorius Stedim, Göttingen, Germany) for 30 min. After normalizing for protein concentrations, immunoblot procedures were performed with the relevant antibodies.

Intracranial U251-E6 and E18 mouse xenograft

U251MG derived cells (2x10^6) cells were suspended in Matrigel/PBS (5 µl) and injected into the right corpus striatum of the brains of 6-8-week-old nude mice using a stereotactic frame. Animals were monitored and sacrificed when neurological signs appeared. Kaplan Meier survival analyses and statistical analyses were performed using GraphPad Prism software (version 3.03).

Intracranial glioma mouse xenograft

Gli36.IL-13Rα2 (2 x 10^5 cells) was stably transduced with lentivirus pWPT-GFP, followed by implantation into the right hemisphere (Bregma (0,0) lateral 2 mm and depth 2.5 mm) of six to eight week old female immunodeficient Balb-C nu/nu mice (Animal Resource Centre). Glioma-bearing mice were sacrificed 21 days post-tumor implantation and immediately perfused through the heart with ice-cold PBS. Tumors were harvested and fixed in 4% PFA and 30% sucrose. Cryosections (10 µm) were analyzed histologically using standard H&E staining. For immunofluorescence staining, cryosections were washed in PBS followed by permeabilization with 0.2% triton X-100 for 10 min room temperature (RT). Blocking of sections was done with 5% BSA and 0.2% triton X-100 (in PBS) for 1 h RT. Sections were then incubated with goat anti-human IL-13Rα2 (R&D Systems; 15 µg/ml), anti-MMP2 (Santa Cruz Biotechnology; 1/100), and anti-vimentin (Epitomics; 1/1000) for 2 h RT. After several washes, slides were incubated with rabbit anti-goat AlexaFluor 594 secondary antibody for 1 h RT. Nuclei was counterstained with DAPI (1 µg/ml) for 5 min RT and then slides were mounted using Slow Fade

(Invitrogen Life Technologies). Slides were visualized using confocal microscopy (LSM 510 Meta; Carl Zeiss, Göttingen, Germany) and images were obtained using either a 40x/0.75 numerical aperture (N.A) Plan-Neofluar or 20x/0.75N.A objective (Carl Zeiss). Similar experiments were performed for Kaplan Meir survival analysis using Gli36, Gli36.IL-13Rα2, Gli36.EGFRvIII and Gli36.IL-13Rα2/EGFRvIII.

Co-Localization Studies

For co-localization studies, naïve cells or cells transfected with full length EGFRvIII or variants were seeded at $8x10^4$ cells in 12-well dish. Following day, cells were fixed in 4% PFA for 10 min RT and permeabilized with 0.1% Triton X-100 for 5 min RT. Cells were first blocked with 5% rabbit serum containing 0.3 M glycine (Invitrogen Life technologies) and PBS with 0.1% tween 20 for 1 h RT. Then, they were incubated with anti-human IL-13Rα2 (R&D Systems; 15 µg/ml) at 4ºC overnight. After several washes, cells were incubated with rabbit anti-goat AlexaFluor 594 secondary antibody (Invitrogen Life Technologies) for 1 h RT. Following few rinses in 0.1% PBT, cells were blocked 2nd time with 5% goat serum containing 0.3 M glycine and PBS with 0.1% tween 20 for 1 h RT. Then cells were incubated with mouse anti-human EGFR (DakoCytomation; 1/75) for 2 h RT. After washes in 0.1% PBT, cells were incubated with goat anti-mouse AlexaFluor 488 for 1 h RT. Nuclei was counterstained with DAPI (1 µg/ml) for 5 min RT and then coverslips were mounted using Slow Fade (Invitrogen Life Technologies).

Immunohistochemistry staining

Brain tissue were fixation with 4% paraformaldehyde (PFA), process and embedded in paraffin. Section slide were subjected to antigen retrieval with sodium citrate buffer, pH 6.0. Endogenous peroxidase was quenched with 0.3% H_2O_2 for 20 min and then blocked with 5% BSA at RT. Primary antibodies used in IHC were as follows: IL-13Ra2 (AF146, R&D Systems), EGFR (MA5-13343, ThermoFisher Scientific). After washing, sections were incubated with either respective secondary antibody before DAB chromogenic detection. The sections were then counterstained with Hematoxylin prior to mounting and visualization.

Immunoblot Analysis

Cells were lysed in lysis buffer (50 mM Tris, 150 mM NaCl, 1% triton X-100) supplemented with fresh protease inhibitor cocktail (Roche), and phosphatase inhibitor (Sigma-Aldrich). For brain tissue specimens, the samples were homogenized with protein extraction buffer (1% Triton X-100, 300 mM NaCl, 2mM EDTA, 400 µM Na_3VO_4, 1% NP-40) supplemented with fresh protease and phosphatase inhibitors. Cell extracts were incubated on ice for 10 min and clarified by centrifugation at 10,000 rpm for 10 min at 4ºC. Protein concentration was determined by Bio-Rad protein assay (Bio-Rad Laboratories, CA, USA). Briefly 40-100 µg of proteins was resolved by 8-15% SDS-PAGE and electroblotted to polyvinylidene fluoride (PVDF) membrane (Trans-Blot Transfer medium; Bio-Rad Laboratories). Membranes were immunoblotted with indicated primary antibodies diluted in blocking buffer. The blocking buffer consists of either PBS with 5% bovine serum albumin (Thermo Scientific) or 5% non-fat milk power in 0.1% PBS consisting of 0.1% tween 20 (Sigma). The rinsed membrane was subsequently incubated with secondary antibodies including goat anti-rabbit, goat anti-mouse immunoglobulin (DakoCytomation; 1/20 000) or rabbit anti-goat immunoglobulin (Santa Cruz Biotechnology; 1/10 000) for 1 h RT. Immunoreactivity was detected with Western Lightning chemiluminescent kit (PerkinElmer). The band density of specific proteins was quantified using either MetaVue (Ver. 6.1) (Molecular Devices Corp, CA, USA) or ImageJ (NIH, MD, USA) software. For all immunoblots, pan-actin served as internal loading controls. The values derived are from either analyzing a single experiment normalizing each of the signaling proteins against the internal control, or as a ratio of phosphorylated form over the total protein. Each of these was performed at least two independent times.

Co-Immunoprecipitation (co-IP)

Human glioma cells were seeded at $2x10^6$ cells per 100mm dish in complete medium. Exponentially growing cells were washed in PBS before crosslinking in 2 mM DSP diluted in PBS for 1 h RT. Crosslinking reaction was stopped with 20 mM Tris, pH 7.5 for 15 min at RT. Subsequently cells were washed again in cold PBS, gently scrapped off and centrifuged at 1000 rpm for 5 min. Cell pellet was then lysed with NP-40 lysis buffer (50 mM Tris-HCl pH8.0, 150 mM NaCl, 1% NP-40) supplemented with protease and phosphatase inhibitors (Roche).

Lysates were homogenized through 23.5 gauge needles, incubated on ice for 15 min and then clarified by centrifugation at 13, 400 rpm for 15 min at 4ºC. Clarified lysates of 1 mg per sample were pre-cleared with non-specific IgG antibody, following by adding protein G Agarose beads (Millipore). The pre-cleared lysates were subsequently incubated with 2 ug of specific antibody (anti-IL-13Rα2, anti-EGFR or anti-Grb2) for 4 h at 4ºC. Thereafter, protein G beads were added to the samples and the reaction mixture was incubated overnight at 4ºC. Following day, after the beads were washed 3 times in ice-cold NP-40 buffer, 2x laemmli buffer was added and heated at 95ºC for 10 min. Both IP and total input samples were loaded unto 8% SDS-PAGE and analyzed by immunoblotting.

Proximity Ligation Assay

Gli36.EGFRvIII cells were transfected with pIRESneo2 encoding either wild-type IL-13Rα2, IL-13Rα2 cytoplasmic deleted mutant or empty vector alone. Twenty four hours post transfection, the cells were seeded on an eight-chamber slide (Thermo Scientific), and incubated for a further 24h prior to fixation and permeabilization in 0.1% Triton X-100 in PBS. Non-specific binding was blocked by incubation with Blocking Solution (Duolink, Sigma Aldrich) for 1hr at RT. Rabbit monoclonal antibody targeting targeting EGFR (Clone D38B1, Cell Signaling Technology) was incubated overnight at 4ºC together with goat antibody targeting IL13Rα2 (AF146, R&D Systems). PLA minus probes against rabbit and plus against goat from (Duolink, Sigma Aldrich) were used in conjunction with Duolink In Situ PLA Red Kit (Duolink, Sigma Aldrich) according to the manufacturer's description. Negative controls were carried out in the absence of primary or secondary antibody while stable clones Gli36.IL-13Rα2/EGFRvIII serve as positive controls. The PLA signals (red), indicative of sites of protein-protein interaction, were observed and the images were captured using under the fluorescence microscope (Olympus, Tokyo, Japan) at 400x magnification. Similar experiments were performed with Gli36.IL-13Rα2 cells transfected with full length EGFRvIII, DK and DY3 mutants.

Ras Activation Assay

Ras is cycling between an inactive GDP-bound form and an active GTP-bound form. In its GTP-bound active state, Ras binds specifically to the Ras-binding domain (RBD) of Raf1 to control downstream signaling cascades. GTP-Bound Active RAS pull-down assay was performed using the Raf-1 RBD agarose beads (Cat#STA-400, Cell Biolabs, CA, USA) which it interacts and precipitates GTP-bound Ras from cell lysate and pull down only the active Ras. Cells were serum starved overnight before harvested for Ras activation assay. Fresh lysates from each cell lines were incubated with 30ul of Raf-1 RBD agarose beads for 3 hours at 4 °C. GTPγS and GDP were loaded to serve as positive and negative control, respectively. The precipitated GTP-Ras was eluted in 2x SDS sample buffer and visualized by immunoblotting with anti–pan-Ras antibodies. The levels of Ras activation are then expressed as a ratio of Ras-GTP to the total Ras/actin levels in the same lysates. Ras activation is also measured using ELISA kit (Cat#STA-440, Cell Biolabs) in accordance to the manufacturer's instruction. Absorbance of each microwell was read on a spectrophotometer using 450 nm as the primary wave length.

RNA isolation and qRT-PCR (Quantitative Reverse Transcription PCR)

Total RNA was isolated using TRIzol Reagent according to the manufacturer's instructions (QIAGEN, CA, USA). Total RNA from samples was reverse transcribed into cDNA using RevertAid First Strand cDNA Synthesis Kit (Thermo Scientific). Real-time PCR was performed on CFX96 Touch™ (Bio-Rad Laboratories) using QuantiTect SYBR Green PCR Kit (QIAGEN). Amplification was done under following conditions: 95ºC for 15min; followed by 40 cycles of 94ºC, 15 sec; 55ºC, 30 sec and 72ºC, 30 sec. GAPDH was use as endogenous control. The $2^{-\Delta\Delta Ct}$ method was used to calculate fold changes in gene expression. Each sample was run in triplicates and at least three experiments were analyzed. Primer sequences used are: GAPDH forward sequence 5'-GAAGGTGAAGGTCGGAGTCA-3'; reverse sequence 5'-TTGAGGTCAATGAAGGGGTC-3', TGFβ forward sequence 5'-CACTCTCAAACCTTTACGAGACC-3'; reverse sequence 5'-CGTTGCTAGGGGCGAAGATG-3'

SUPPLEMENTARY FIGURES

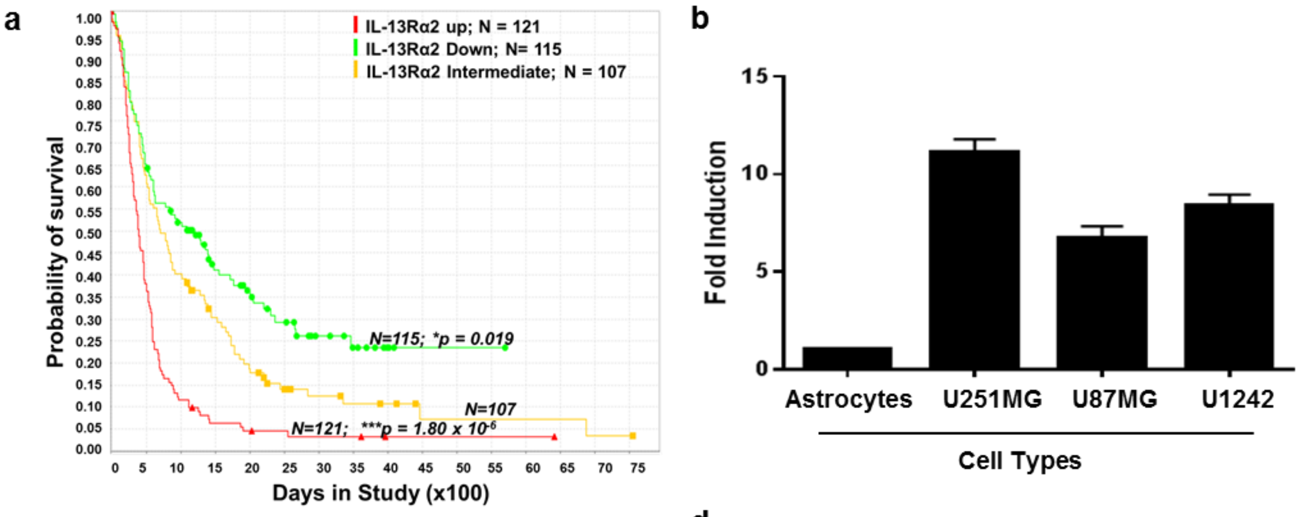

c

	Chi-Square Table			N
	IL13Rα2/ Hi	IL13Rα2/ Intermediate	IL13Rα2/ Low	
EGFR/Hi	94<br>96.66<br>(0.07)	78<br>85.48<br>(0.65)	102<br>91.87<br>(1.12)	**274**
EGFR/ Intermediate	21<br>20.11<br>(0.04)	25<br>17.78<br>(2.93)	11<br>19.11<br>(3.44)	**57**
EGFR/Low	6<br>4.23<br>(0.74)	4<br>3.74<br>(0.02)	2<br>4.02<br>(1.02)	**12**
N	**121**	**107**	**115**	**343**

$\chi^2 = 10.030$, df = 4, $\chi^2/df = 2.51$, $P(\chi^2 > 10.030) = 0.0399$

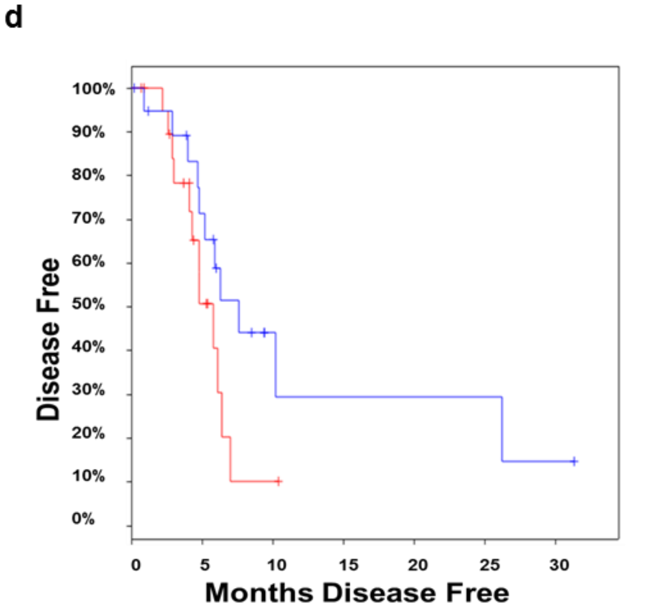

Supplementary Figure 1

Supplementary Figure 1

Analysis of EGFR and IL-13Rα2 expression in Glioma.

(**a**) Kaplan-Meier survival plots derived from REMBRANDT database from National Cancer Institute (USA) showing glioma samples with differential levels of IL-13Rα2 expression. Of the 343 patients evaluated, 121 patients expressing high levels of IL-13Rα2 (red) showed statistically significant decreased survival compared to the intermediate IL-13Rα2 (log-rank ****p* value <0.01). In contrast, the 115 patients expressing low levels of IL-13Rα2 (green) showed improved survival outcome when compared to the same intermediate group (log-rank * *p* value <0.05). (**b**) RT-PCR expression of IL-13Rα2 in glioma cell lines versus normal astrocytes. (**c**) The *chi square analysis* by SAS biostatistical software show an association between EGFR and IL-13Rα2 using all glioma patient dataset from Rembrandt database (https://caintegrator.nci.nih.gov/rembrandt/). (**d**) High expression of IL-13Rα2 together IL-13 had negative impact on patient survival by TCGA analysis.

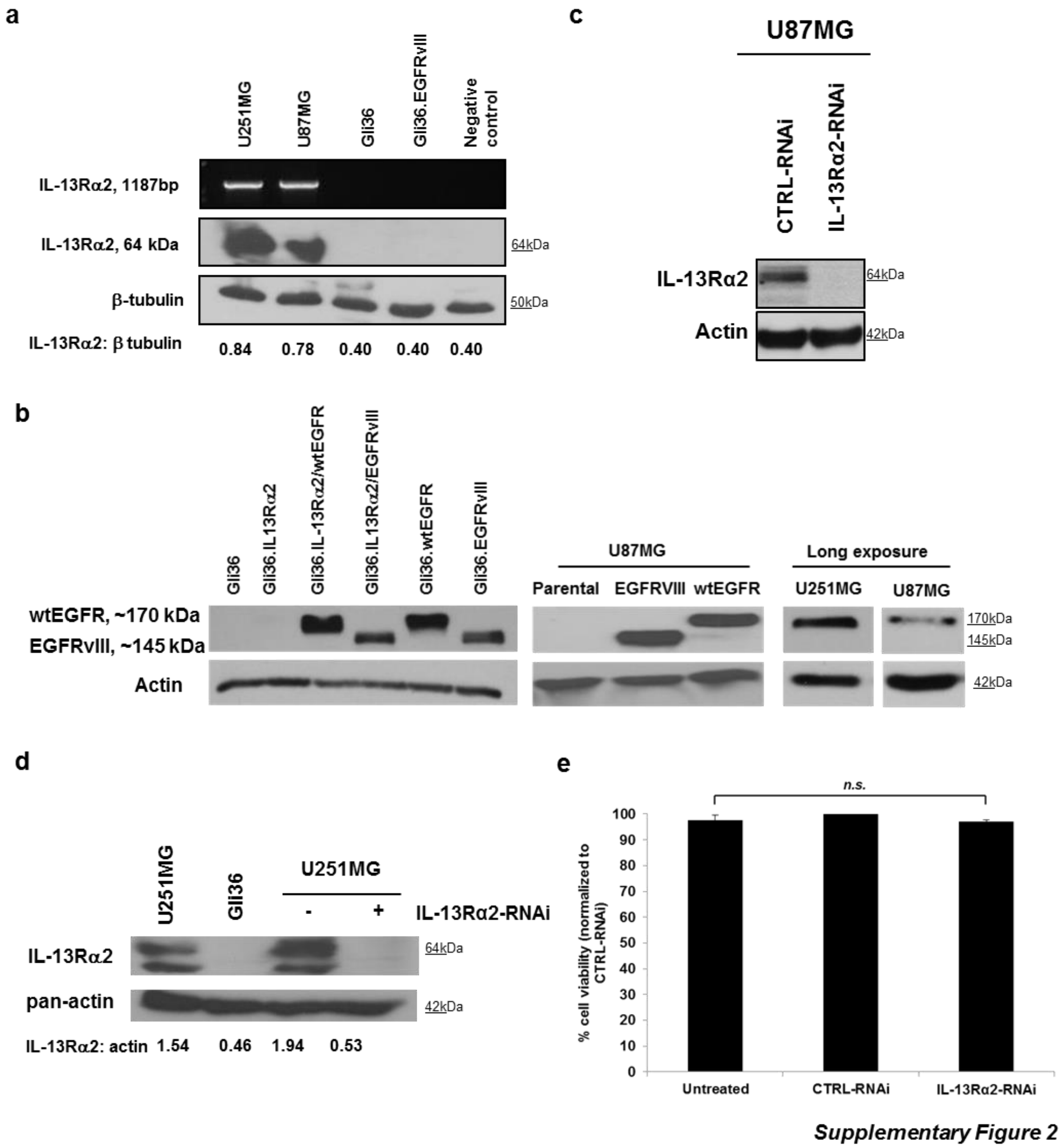

Supplementary Figure 2

IL-13Rα2 knockdown abolishes glioma cell invasion but does not affect cellular proliferation.

(**a**) Expression of endogenous IL-13Rα2 was examined in a series of human glioma cell lines by RT-PCR and immunoblot analysis. Ratios represents densitometry quantification normalized to either β-tubulin or pan-actin as seen appropriate. No template control served as negative control for RT-PCR. (**b**) The status of EGFR expression was determined in Gli36 and U87MG isogenic cell lines, as well as in U251MG cells by immunoblot analysis. A long exposure was also performed for U87MG indicating a low level of endogenous EGFR could also be detected. Pan-actin was used as loading control. Validation of targeted knockdown of IL-13Rα2 in (**c**) U87MG cells and in (**d**) U251MG cells at 48 h post-RNAi transfection. (**e**) Cell viability was determined with trypan blue exclusion assay and percentage viable cells were normalized to the untreated U251MG cells. All data are represented as mean ± SEM. *p<0.05; ***p* < 0.01; *n.s.* not significant.

f

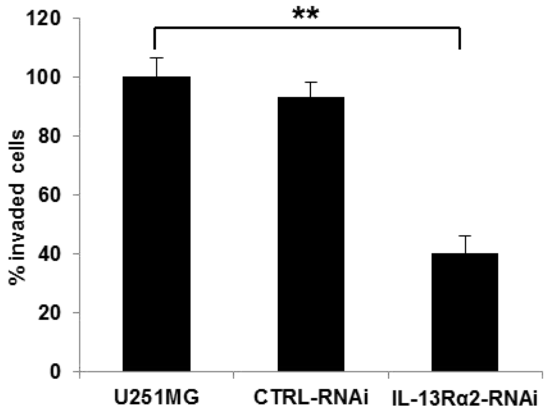

g

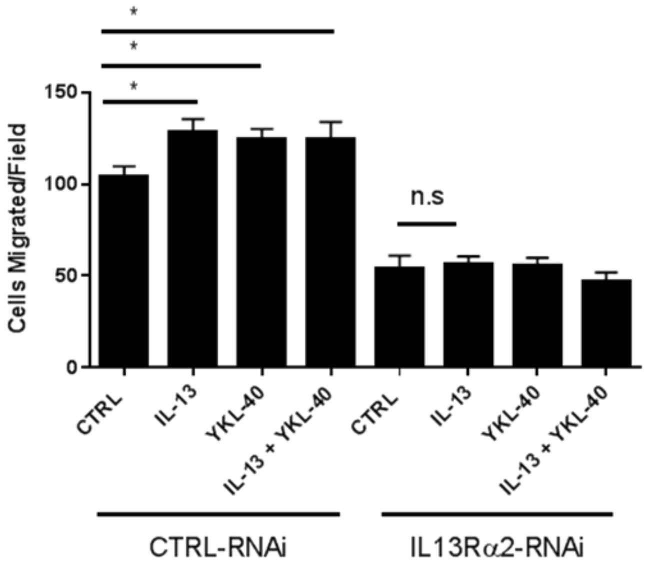

h

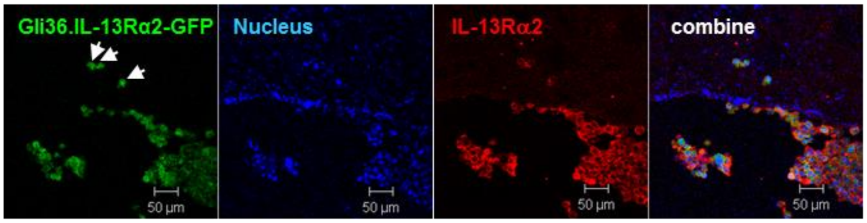

Supplementary Figure 2

Supplementary Figure 2 (cont.)

(**f**) Invasive capacity of IL-13Rα2 knockdown was determined and normalized to untreated U251MG cells. (**g**) *In vitro* migratory capacity of control and IL-13Rα2-RNAi treated U251MG cells, stimulated with 1 µg/ml YKL40 or 20 ng/ml IL-13 for 18h, was determined using wound-healing migration assay. (**h**) Confocal representative images of EGFRvIII (green) and IL-13Rα2 (red) in Gli36.IL-13Rα2/EGFRvIII cells. Nucleus is counterstained by DAPI (blue). Scale Bar, 50 µm.

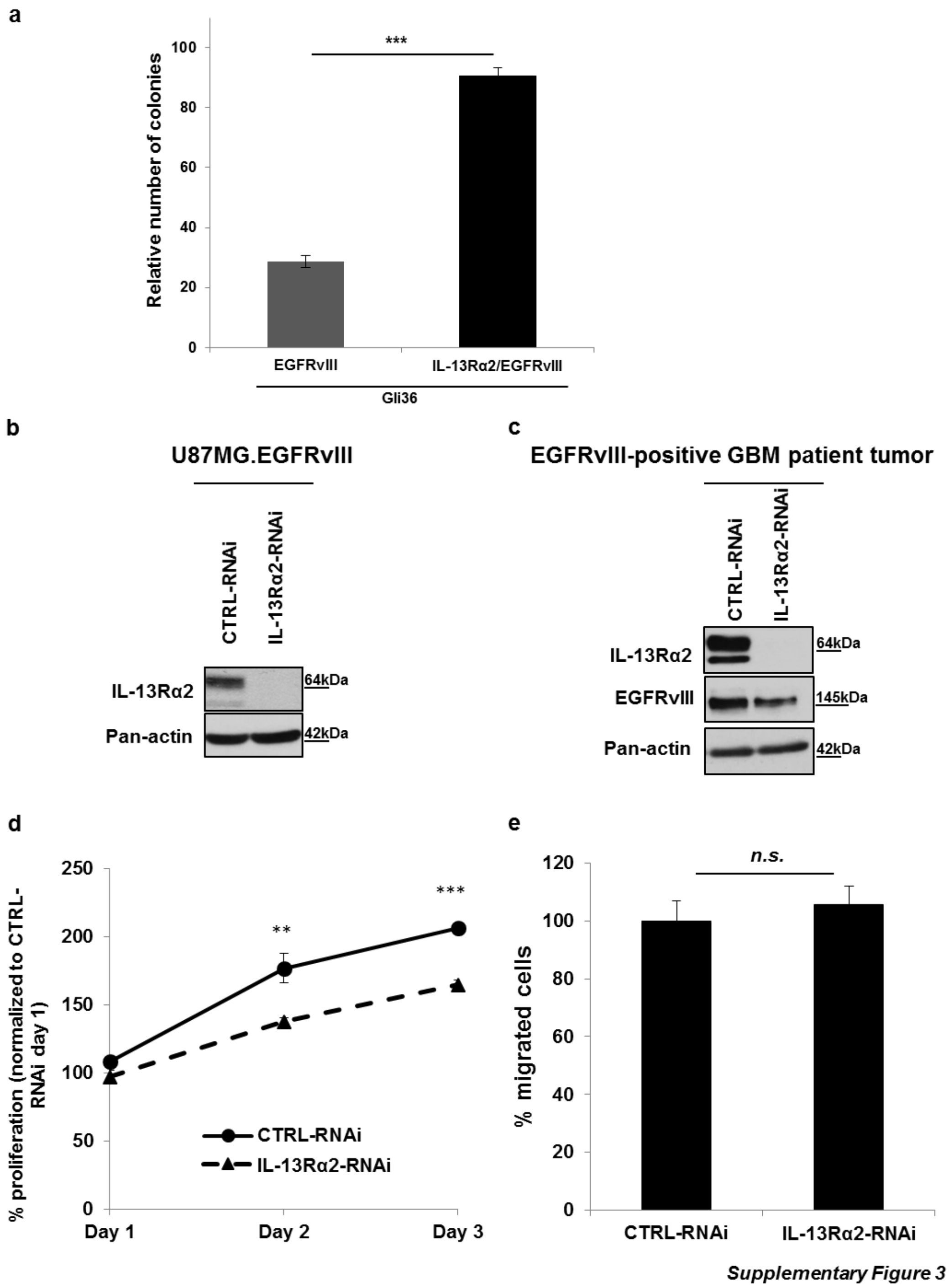

Supplementary Figure 3

Co-expression of IL-13Rα2 and EGFRvIII receptors in glioma cells confers growth advantage

(**a**) Soft agar colony formation assays were performed in Gli36.EGFRvIII versus Gli36.IL13Rα2/EGFRvIII cells. Relative number of colonies was determined by counting the number of colonies in each plate. Bar graph represents mean ± SEM, ***$p < 0.001$. Immunoblotting validation of targeted knockdown of IL-13Rα2 in (**b**) U87MG.EGFRvIII cells and (**c**) EGFRvIII-positive GBM patient tumor derived from Mayo clinic at 48h post RNAi transfection. Targeted knockdown of IL-13Rα2 in EGFRvIII-positive GBM patient tumor resulted in reduced (**d**) cell proliferation but there was no difference in (**e**) cell migration.

f

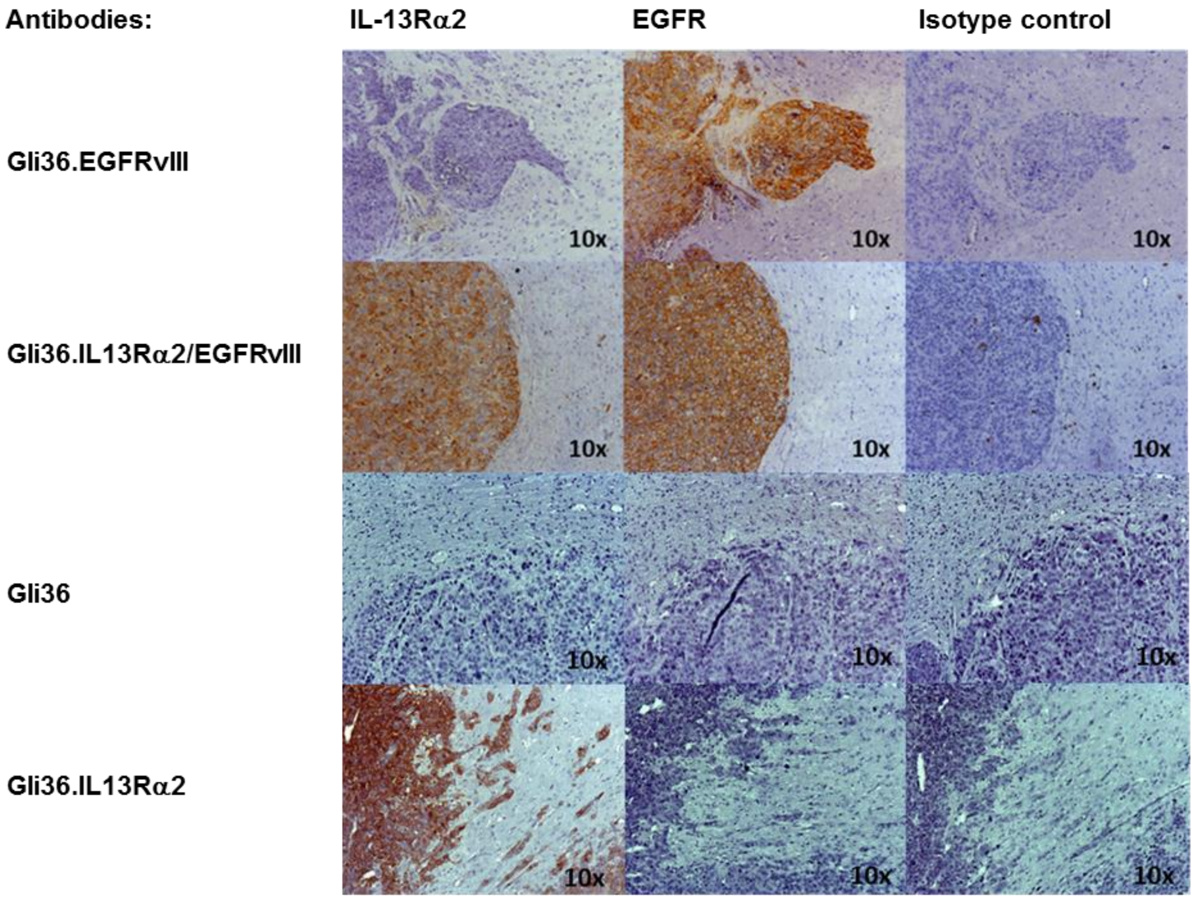

Supplementary Figure 3

Supplementary Figure 3 (Cont.)

(**f**) Immunohistochemistry staining confirming the expression of EGFR and IL-13Rα2 expression in Gli36.EGFRvIII, Gli36.IL-13Rα2/EGFRvIII cells, Gli36 and Gli36.IL-13Rα2 brain tumors harvested from representative animals from the Kaplan Meier survival study.

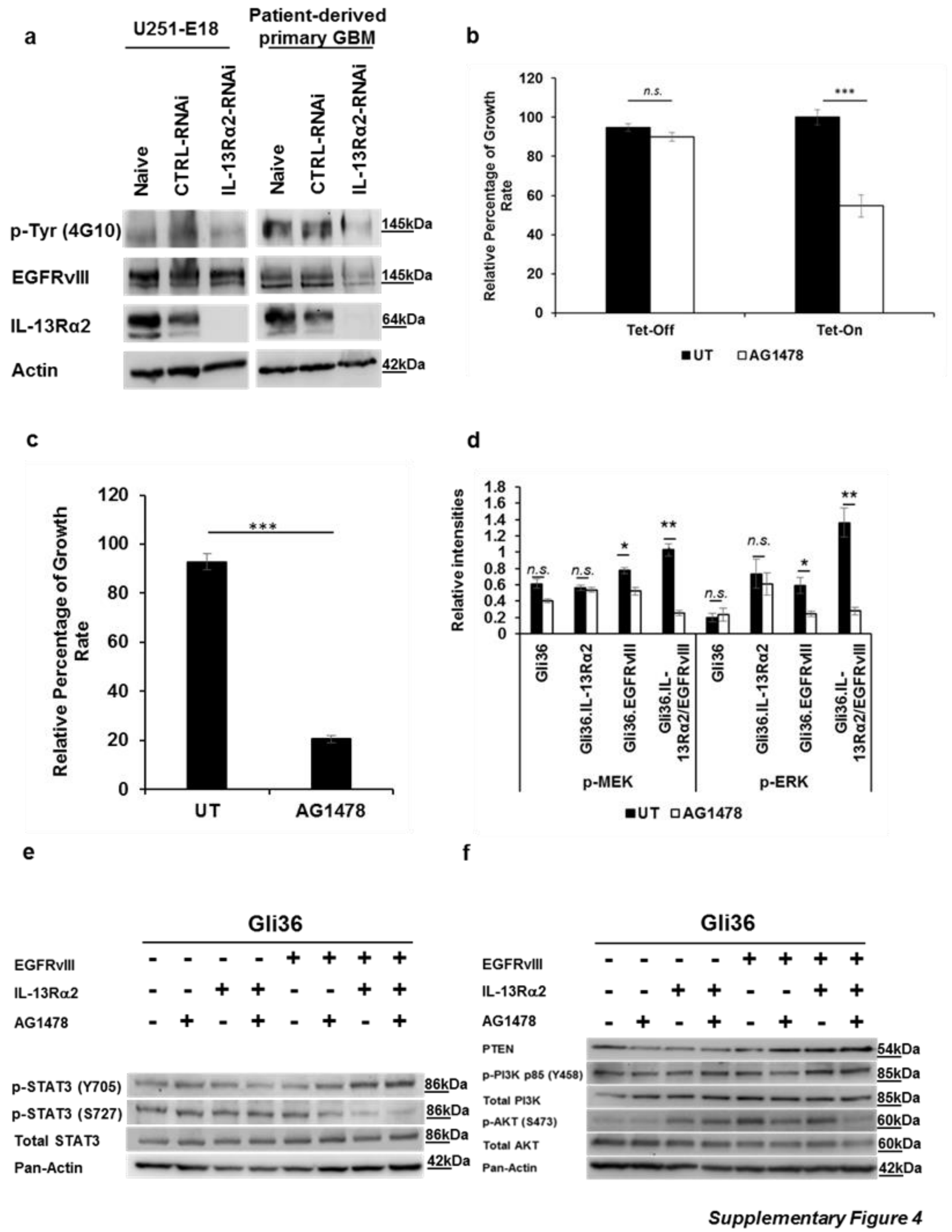

Supplementary Figure 4

Enhanced tyrosine kinase activites and cell proliferation are mediated by oncogenic signaling of IL-13Rα2 and EGFRvIII through activated RAS/RAF/MEK/ERK signaling.

(**a**) Cell lysates from transient knockdown of IL-13Rα2 in U251-E18 or patient-derived primary GBM were examined for the total levels of tyrosine phosphorylation using anti-phosphotyrosine antibodies. Experiments performed from (**b-h)** were in the presence of 10μM AG1478 treatment or without. CCK-8 proliferation assay was performed in (**b**) U251-E18 cells and (**c**) EGFRvIII-positive patient-derived primary GBM cells. (**d**) Densitometry semi-quantification of phosphorylated MEK and ERK of indicated cell lines with and without AG1478. Endogenous expression levels of (**e**) total and p-STAT3 and (**f**) PTEN, total and p-PI3K p85α, total and p-AKT were examined in indicated cell lines under different conditions.

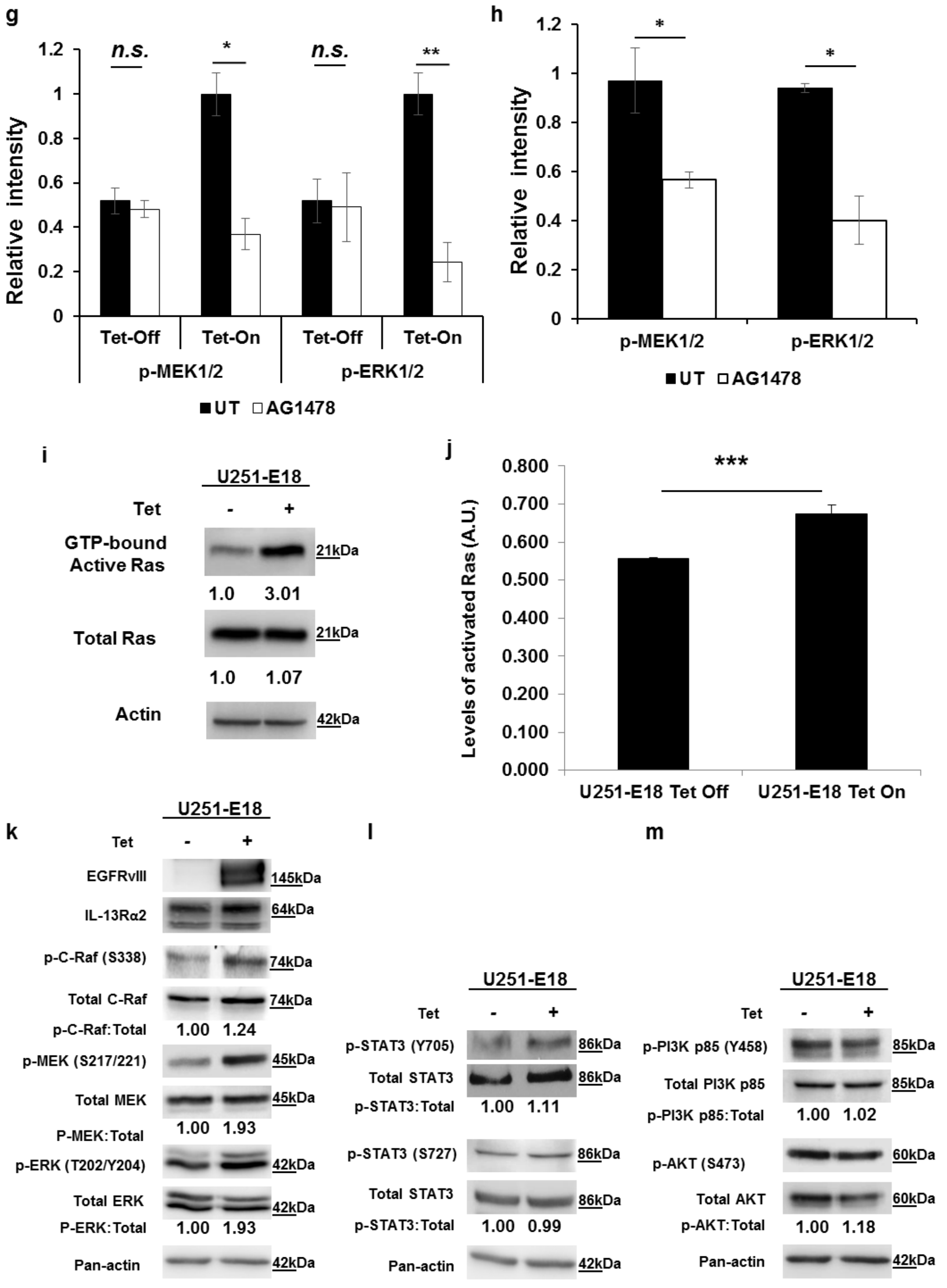

Supplementary Figure 4

Supplementary Figure 4 (Cont.)

Densitometry semi-quantification of phosphorylated MEK and ERK levels in (**g**) U251-E18 and (**h**) EGFRvIII-positive patient-derived primary GBM cells. RAS activation in U251-E18 cells was determined by (**i**) Raf-1 RBD agarose beads pull down assay or (**j**) ELISA. Endogenous expression levels of (**k**) total and p-C-RAF, total and p-MEK/p-ERK, (**l**) total and p-STAT3, (**m**) PTEN, total and p-PI3K p85α, total and p-AKT were examined in U251-E18 cells.

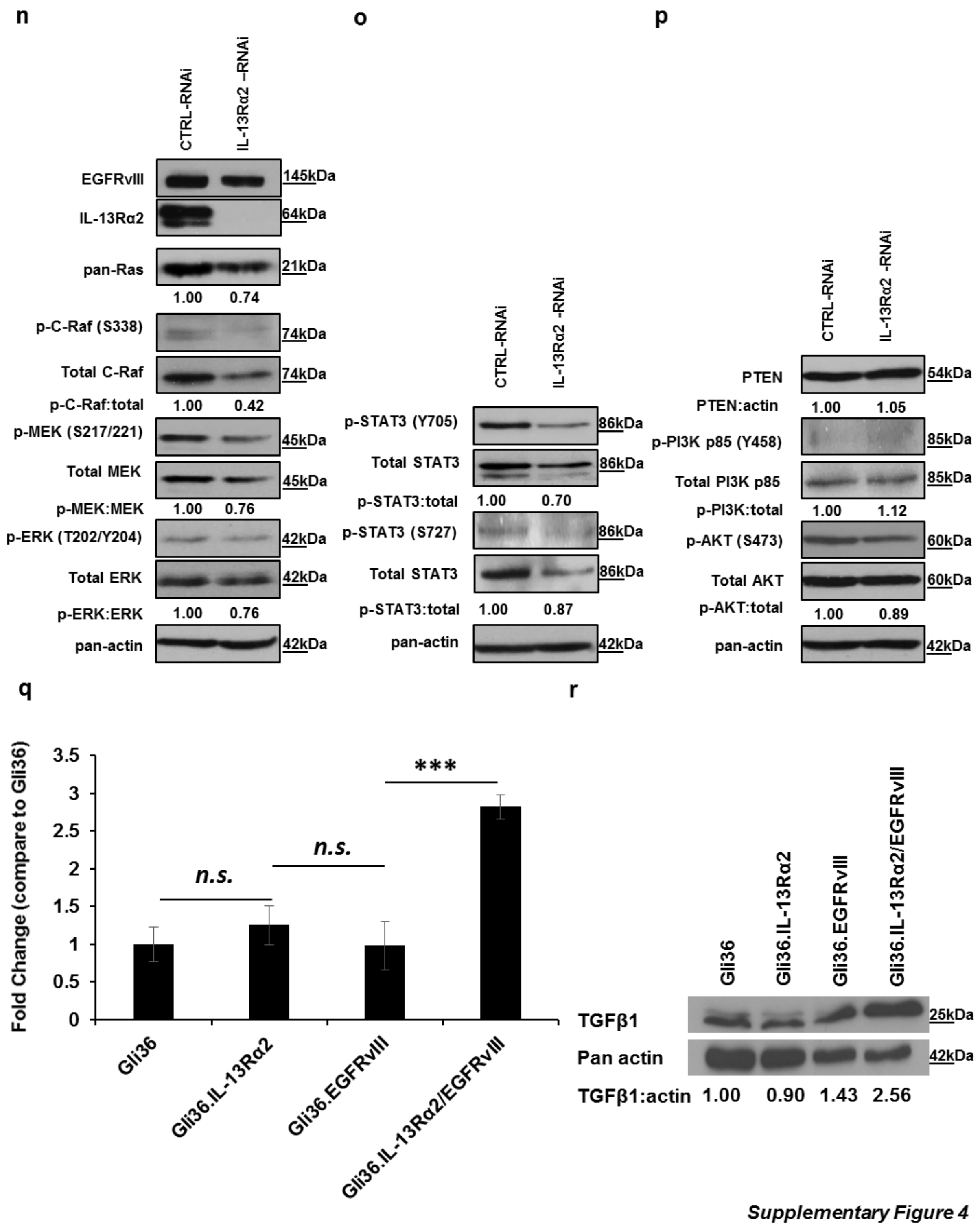

Supplementary Figure 4 (Cont.)

Targeted knockdown of IL-13Rα2 in patient-derived primary GBM from Mayo clinic that express endogenous EGFRvIII and IL-13Rα2 resulted in significant reduced levels of (**n**) Pan-RAS, Total and p-C-RAF, Total and p-MEK/p-ERK and (**o**) total and p-STAT3 expressios while there was no significant change in the levels of (**p**) PTEN, total and p-PI3K p85α and total and p-AKT. Transcription and endogenous protein express levels of TGFβ were examined by (**q**) qRT-PCR and (**r**) immunoblot analysis in the indicated cell lines.

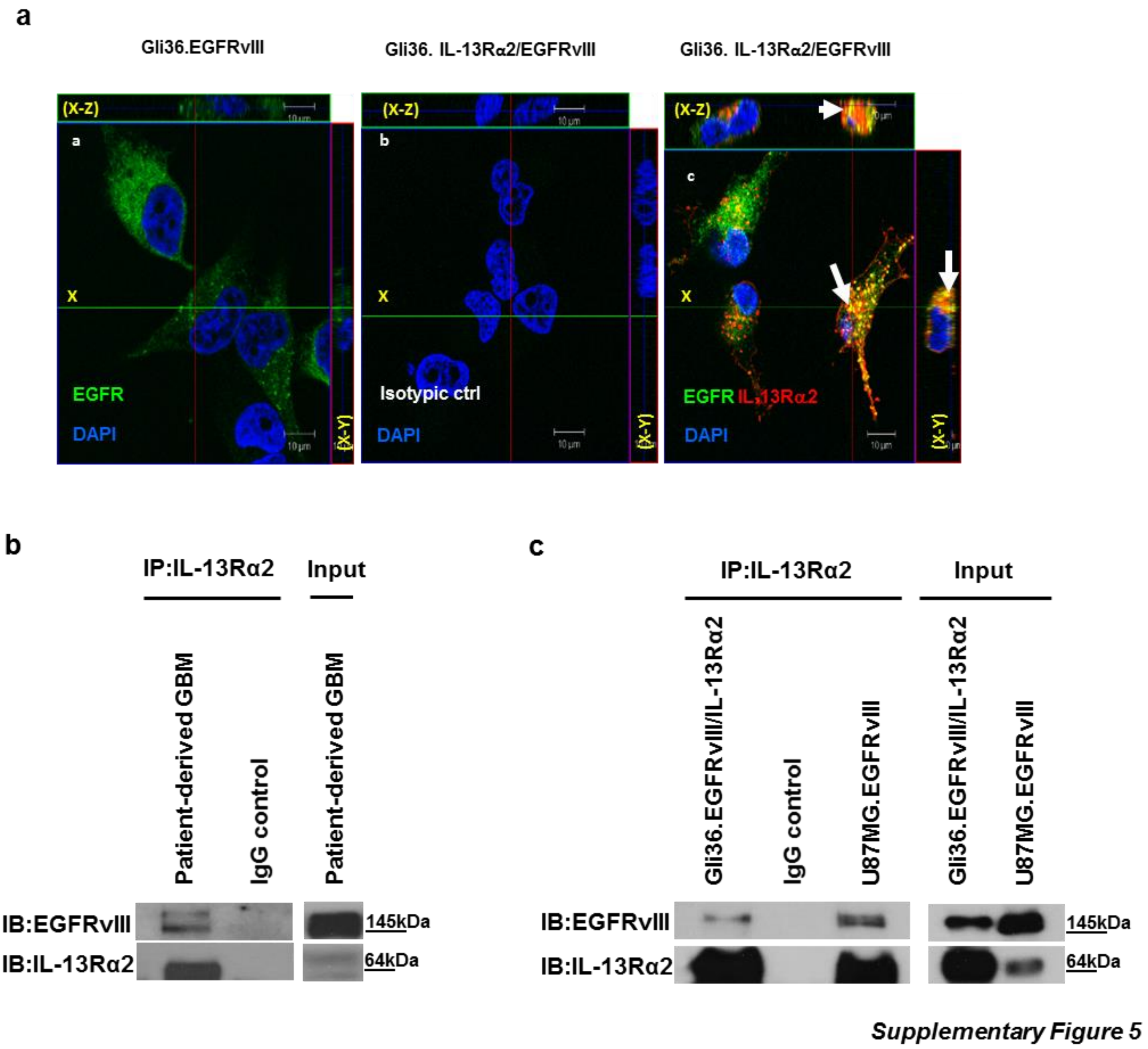

Supplementary Figure 5

Enhanced growth is mediated through specific interaction of IL-13Rα2 with EGFRvIII

Confocal studies were performed in the isogenic glioma cells. The cells were incubated with anti-IL-13Rα2 alone, EGFR alone and isotypic control served as controls. (**a**) Subcellular localization of EGFRvIII (green) and IL-13Rα2 (red) in Gli36.IL-13Rα2/EGFRvIII cells. Co-localization of both proteins is observed in the cytosol as indicated by white arrows. Nucleus is counterstained by DAPI (blue). Scale Bar, 10 µm. Images were obtained with a confocal system (LSM 510 Meta; Carl Zeiss, Göttingen, Germany) using a 100x/1.3N.A. Plan NeoFluar oil immersion objective (Carl Zeiss). Co-immunoprecipitation experiments were performed in (**b**) primary EGFRvIII-positive GBM patient tumor derived from Mayo clinic, and in (**c**) glioma cells expressing endogenous IL-13Rα2 (U87MG.EGFRvIII) where Gli36.IL-13Rα2/EGFRvIII served as positive control.

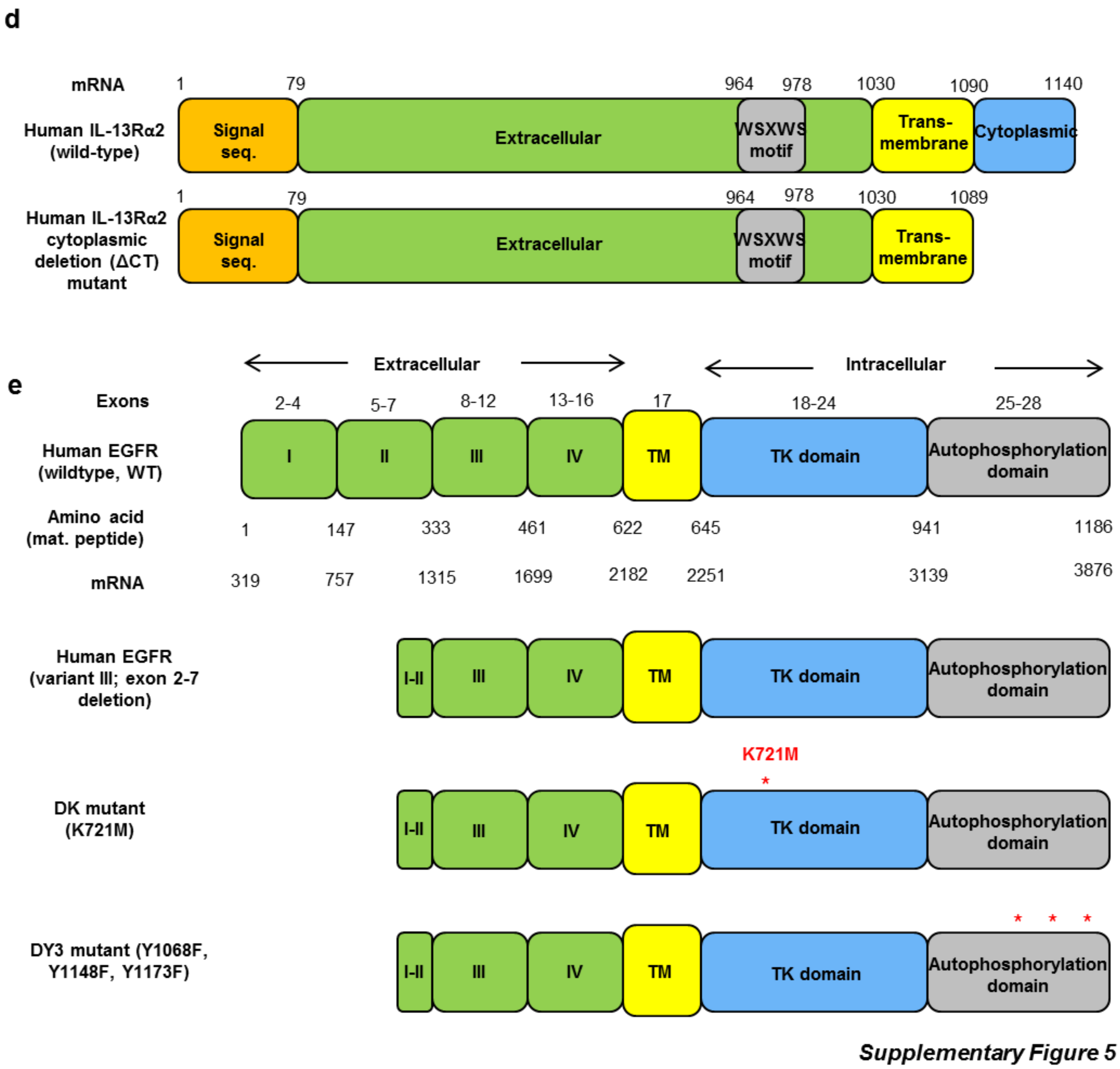

Supplementary Figure 5 (Cont.)
(**d**) Representative diagrams of human IL-13Rα2 also denoted as wild-type and IL-13Rα2 Cyt tail deleted (ΔCyt tail) mutation. (**e**) Representative diagrams of human EGFR, also denoted as wild-type (WT) EGFR, EGFRvIII, EGFRvIII kinase dead (K721M), EGFRvIII site mutations (Y1068F, Y1148F, Y1173F).

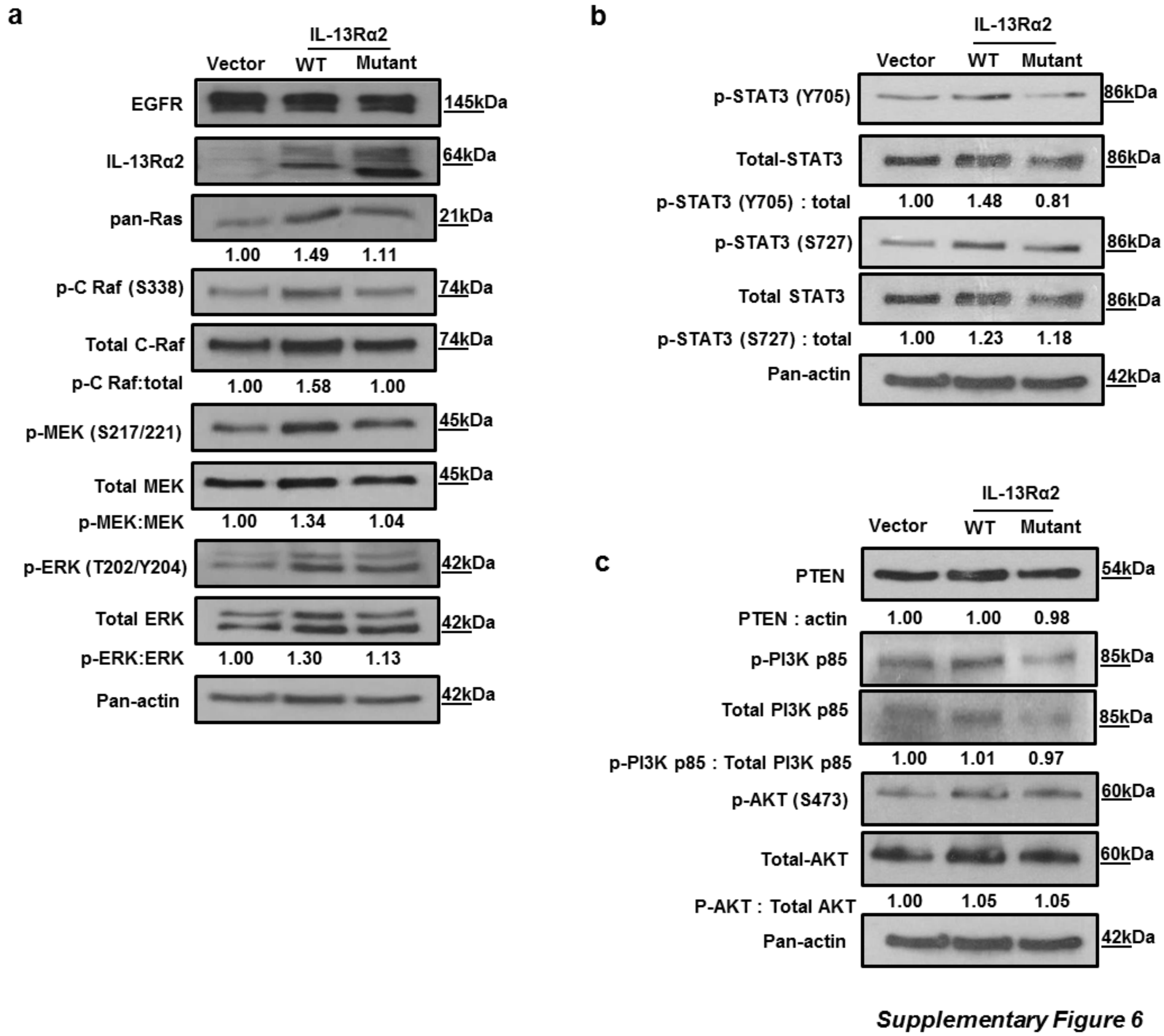

Supplementary Figure 6

Deletion of the cytoplasmic domain of IL-13Rα2 resulted in the loss of elevated MAPK activities

To further examine the domains of interaction, Gli36.EGFRvIII cells were first transfected with pIRESneo2 vector, IL-13Rα2 full length (Wild-type) and IL-13Rα2 Cyt tail deleted constructs (Mutant). Cell lysates were harvested and analyzed against key signaling proteins in (**a**) MAPK, (**b**) STAT3, (**c**) PTEN, PI3K/AKT by immunoblotting assays. For all immunoblots, pan-actin served as internal loading controls, and band densitometry quantifications for the proteins were performed using ImageJ (NIH). The value derived is obtained from normalizing each of the signaling proteins against actin in a single experiment, and presented as a ratio of phosphorylate form over total protein.

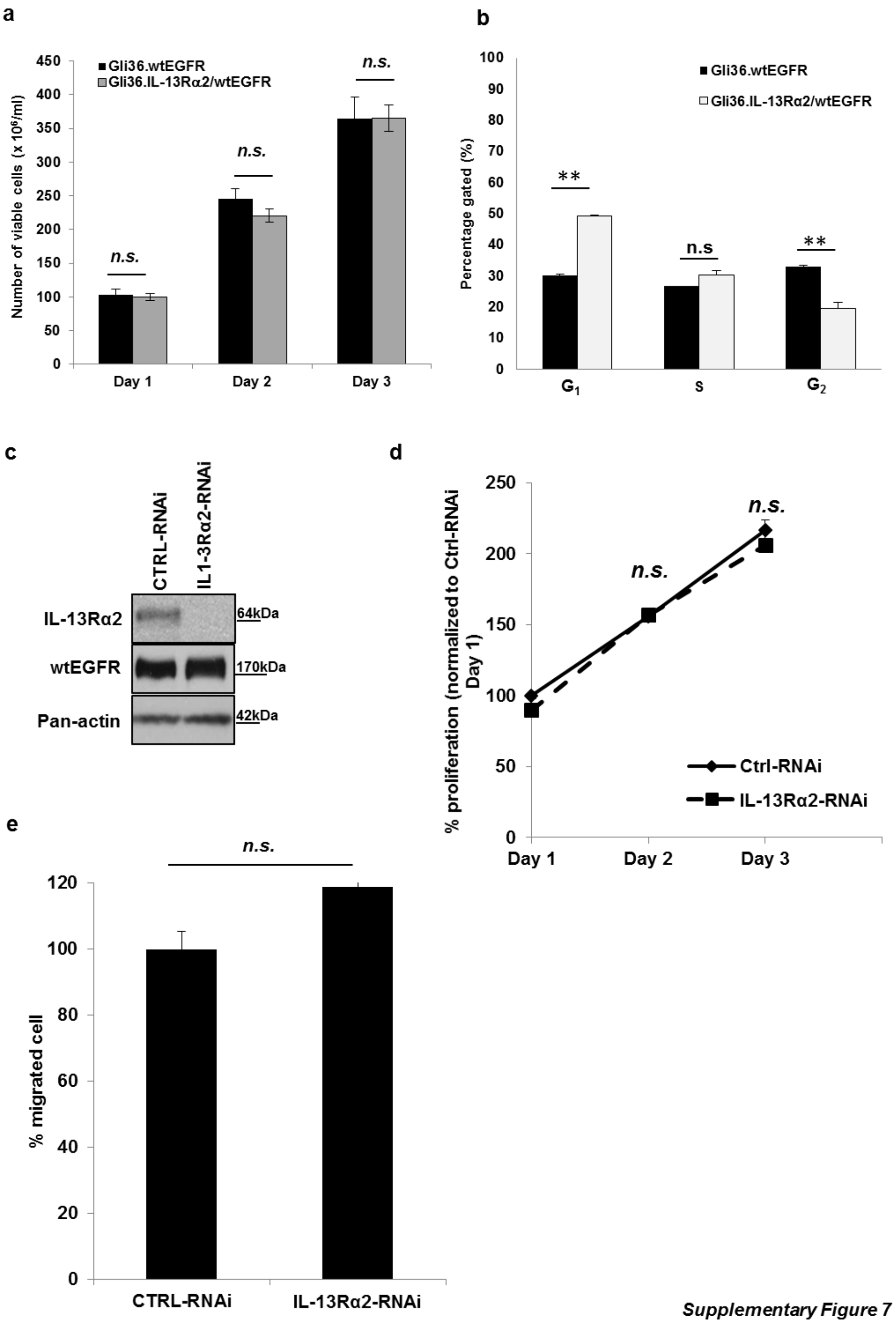

Supplementary Figure 7

Co-expression of IL-13Rα2 and wtEGFR receptors in glioma cells did not confer growth advantage

(**a**) Cell proliferation and (**b**) cell cycle analysis were performed with Gli36.wtEGFR and Gli36.IL-13Rα2/wtEGFR cells using CCK-8 proliferation assay and flow cytometry respectively. All data are represented as mean ± SEM. **$p < 0.01$; *n.s.* not significant. (**c**) Immunoblotting validation of targeted knockdown of IL-13Rα2 in wtEGFR-positive GBM patient tumor derived from Mayo clinic at 48h post RNAi transfection. Targeted knockdown of IL-13Rα2 in wtEGFR-positive GBM patient tumor resulted in no difference in (**d**) cell proliferation and in (**e**) cell migration.

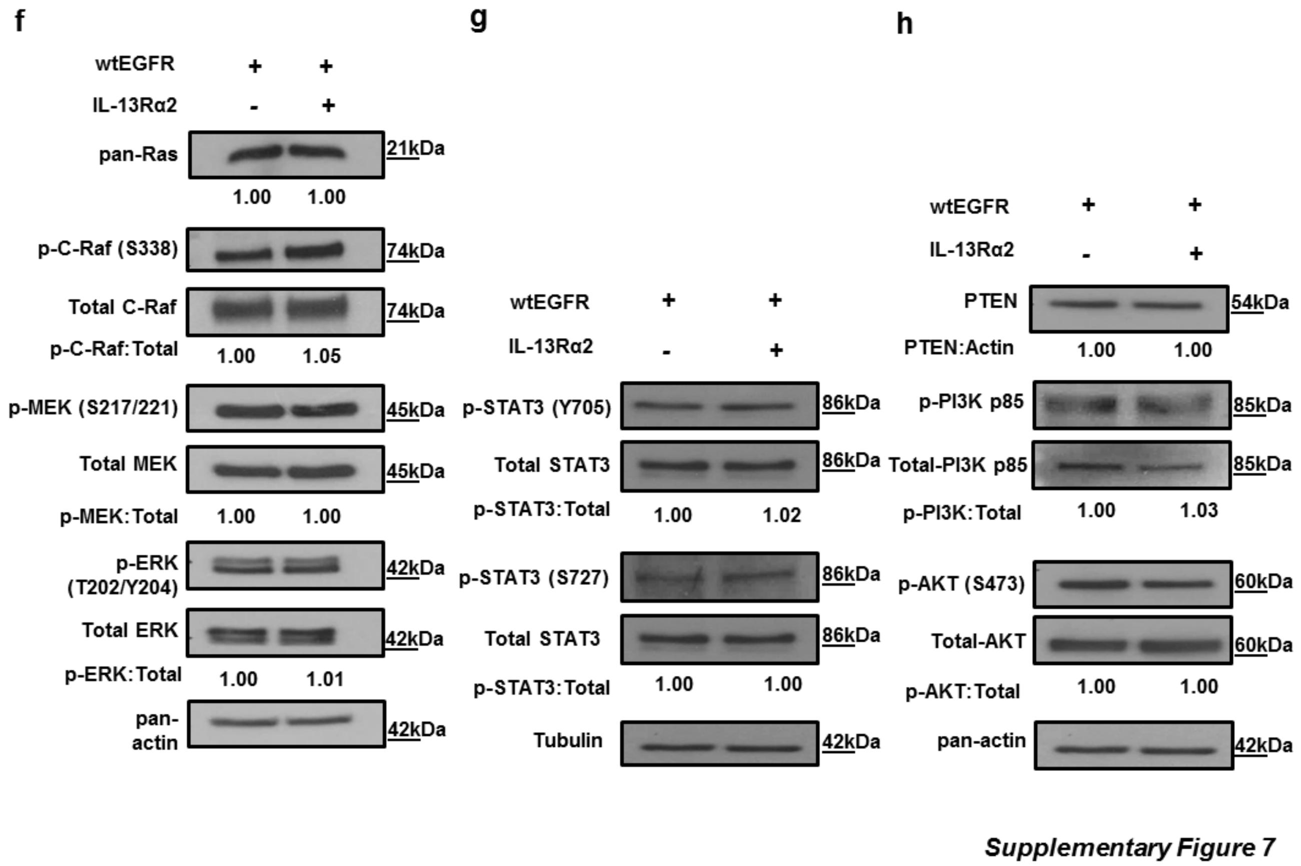

Supplementary Figure 7 (Cont.)
Key signaling pathways in Gli36.wtEGFR and Gli36-IL-13Rα2/wtEGFR cells were examined by immunoblotting of endogenous expression levels of (**f**) MAPK, (**g**) STAT3 and (**h**) PTEN/PI3K/AKT pathway.

References

1. Ramnarain DB, *et al.* Differential gene expression analysis reveals generation of an autocrine loop by a mutant epidermal growth factor receptor in glioma cells. *Cancer Res* **66**, 867-874 (2006).

2. Chong DQ, *et al.* Combined treatment of Nimotuzumab and rapamycin is effective against temozolomide-resistant human gliomas regardless of the EGFR mutation status. *BMC Cancer* **15**, 255 (2015).
